# Evolutionary Analyses of *Staphylococcus aureus* Identify Genetic Relationships between Nasal Carriage and Clinical Isolates

**DOI:** 10.1371/journal.pone.0016426

**Published:** 2011-01-21

**Authors:** Ryan P. Lamers, Jason W. Stinnett, Gowrishankar Muthukrishnan, Christopher L. Parkinson, Alexander M. Cole

**Affiliations:** 1 Department of Molecular Biology and Microbiology, University of Central Florida College of Medicine, Orlando, Florida, United States of America; 2 School of Electrical Engineering and Computer Science, University of Central Florida, Orlando, Florida, United States of America; 3 Department of Biology, University of Central Florida, Orlando, Florida, United States of America; National Institutes of Health, United States

## Abstract

Nasal carriage of *Staphylococcus aureus* has long been hypothesized to be a major vector for the transmission of virulent strains throughout the community. To address this hypothesis, we have analyzed the relatedness between a cohort of nasal carriage strains and clinical isolates to understand better the genetic conformity therein. To assess the relatedness between nasal carriage and clinical isolates of *S. aureus*, a genetic association study was conducted using multilocus sequence typing (MLST) and typing of the hypervariable regions of clumping factor and fibronectin binding protein genes. At all loci analyzed, genetic associations between both nasal carriage and clinical isolates were observed. Computational analyses of MLST data indicate that nasal carriage and clinical isolates belong to the same genetic clusters (clades), despite differences in sequence type assignments. Genetic analyses of the hypervariable regions from the clumping factor and fibronectin binding protein genes revealed that not only do clinically relevant strains belong to identical genetic lineages as the nasal carriage isolates within our cohort, but they also exhibit 100% sequence similarity within these regions. The findings of this report indicate that strains of *S. aureus* being carried asymptomatically throughout the community via nasal colonization are genetically related to those responsible for high levels of morbidity and mortality.

## Introduction

*Staphylococcus aureus* is a prevalent human pathogen of increasing concern to public health worldwide. This pathogen is one of the leading causes of hospital-acquired infection, and additionally leads to significant levels of infection via community transmission. Approximately 20–30% of the global population is persistently colonized with *S. aureus* in the anterior nares, with 60–100% of individuals projected to be transiently colonized at some point during their lives [1]. Though nasal carriage of *S. aureus* is hypothesized to be a major vector for transmission throughout hospitals and the community, neither the determinants of nasal colonization nor the role of carriage in the propagation of *S. aureus* infection throughout these settings are well established [2].

Multiple studies have shown that nasal carriage of *S. aureus* is a risk factor for pathogenic infection [3], [4], but just recently was it observed that heightened risk is only evident in persistent nasal carriers whereas intermittent and non-carriers exhibit low levels of infection [1]. Aside from straightforward incidences in which endogenous strains establish pathogenic infections in their hosts, the overall extent to which nasal carriage strains are responsible for transmissible infection is not currently known.

Population structure and genetic diversity of *S. aureus* has been extensively studied in the past using pulse-field gel electrophoresis (PFGE) and multilocus sequence typing (MLST) [5], [6], [7], [8], [9], [10]. While PFGE provides adequate strain resolution, it encounters difficulty in reproducing and comparing data between laboratories. Thus, MLST is the primary means by which *S. aureus* strains have been analyzed for the past decade. Yet, because of the slow rate of molecular evolution within MLST genes, this methodology is most useful on a global epidemiology scale [6], [11], [12]. When local investigations are carried out, where a greater level of strain resolution is desired (e.g. local applications such as patient to patient transmission), analyses of hypervariable virulence genes is required [13]. Moreover, recent interest in sub-classifying sequence types (STs) has also identified virulence genes (e.g. clumping factor and fibronectin binding protein gene families) as appropriate targets for obtaining high levels of strain resolution [6], [7]. In addition to their hypervariability, virulence genes are also attractive targets for the assessment of strain pathogenicity since these genes contribute to the invasiveness of the bacterium.

Previous studies that have focused on virulence genes have typically done so in large cohorts of clinical strains where methodologies such as amplified fragment length polymorphism typing, *spa* typing, or double locus sequence typing have been employed [6], [7], [11], [13], [14], [15]. Few studies have analyzed virulence genes to examine *S. aureus* within the community, or to identify the genetic relationships between nasal carriage isolates and those isolated from the clinical setting. Previous studies have identified that most *S. aureus* strains, both nasal and clinical, belong to five major clonal complexes (CCs); CC5, CC8, CC22, CC30, and CC45 [15]; however, it is not well established whether the genes responsible for the pathogenicity of *S. aureus* are genetically similar between clinical and nasal carriage isolates.

Here we have performed evolutionary analyses on the seven MLST gene fragments, as well as the hypervariable regions of virulence genes in a cohort of *S. aureus* nasal carriage strains to analyze the genetic diversity present therein. Contrary to previous reports, we observe higher levels of nucleotide diversity among nasal carriage strains than those for clinical isolates. In addition to analyzing the genetic diversity in our cohort of nasal carriage strains, we also performed a genetic comparison between these strains and strains of clinical significance. We find that both nasal carriage strains from our cohort and clinical strains isolated from symptomatic patients around the world exhibit the same genetic makeup in housekeeping and virulence genes.

## Materials and Methods

### Ethics statement for collection of nasal carriage isolates

Nasal carriage isolates of *S. aureus* were collected from willing donors following University of Central Florida Institutional Review Board (IRB)-approved procedures. Written informed consent was obtained for all donors throughout the study. All study coordinators involved in the sample collection process were IRB-approved with Collaborative Institutional Training Initiative (CITI) certification.

### Bacterial isolates

Two hundred and twenty-two healthy individuals at the University of Central Florida (Orlando, Florida, USA) were prescreened for the presence of *S. aureus* in their nares. Of these, nasal carriage isolates were obtained from 56 (25.2%) individuals and utilized for genetic analyses in this study. Isolates were collected by inserting a single cotton swab into each of a donor's nostrils and circulating for approximately five to ten seconds. As part of an ongoing longitudinal study, we obtained multiple samples from repeat donors, at a minimum interval of one month, to monitor the population genetics of *S. aureus* over time. Thus, our data are a reflection of one representative strain from all individuals involved in the current study, unless multiple samplings identified different strains from the same individual. In those cases, all different strains from one individual were analyzed. Nasal samples were plated on Trypticase^TM^ Soy Agar (TSA) containing 5% sheep's blood (Becton, Dickinson and Company, Franklin Lakes, New Jersey, USA), and incubated at 37°C for 16 hours. Bacterial colonies were identified as *S. aureus* using Staphyloslide^TM^ Latex Test reagent (Becton, Dickinson and Company, Franklin Lakes, New Jersey, USA), and positive colonies were inoculated in 5 mL of Trypticase Soy Broth and grown for 16 hours at 37°C and 250 rpm. Following inoculation, 1.5 mL of bacterial culture was pelleted by centrifugation for two minutes at 16 000× g and culture medium was discarded. Pellets were then stored at −80°C until DNA isolation.

Twenty-eight clinical isolates of *S. aureus* were also utilized in this study to determine the evolutionary relationships between clinical strains and strains present in the nasal carriage population. Gene sequences from 15 clinical isolates with complete genomes available were obtained from the NCBI nucleotide database (http://www.ncbi.nlm.nih.gov/nucleotide/). The previously sequenced clinical strains were N315, Mu50, COL, MRSA252, MSSA476, MW2, USA300_FPR3757, NCTC8325, JH1, JH9, Newman, Mu3, USA300_TCH1516, 04-02981, and TW20 (Table S1). Thirteen additional clinical strains for which clumping factor A (*clfA*), clumping factor B (*clfB*), and fibronectin binding protein A (*fnbA*) repeat region sequences are available on the NCBI nucleotide database were also utilized in this study (Table S1) [13]. Refer to supplemental Table S2 for accession numbers to all DNA sequences utilized in this study.

### DNA isolation/amplification

*S. aureus* genomic DNA was isolated using GenEluteTM Bacterial Genomic DNA kit (Sigma-Aldrich Co., St. Louis, Missouri, USA), according to the manufacturer's instructions. Following DNA isolation, extracts were quantified and stored at −20°C until DNA amplification.

Amplification of multilocus sequence typing (MLST) gene fragments was carried out using primers and protocols described previously [10]. Briefly, 402–516 bp fragments for the seven MLST housekeeping genes (*arcC*, *aroE*, *glpF*, *gmk*, *pta*, *tpi*, and *yqiL*) were amplified and sequenced (see below). Sequence types (STs) were determined for each strain based on the alleles identified at each of the seven loci using the *S. aureus* MLST database (http://www.mlst.net) (Table S1). For instances in which new alleles, or combinations of alleles (i.e. new STs), were identified the MLST database curator was contacted and new allele numbers and STs were obtained.

For *clfA, clfB, fnbA,* and *fnbB*, the repeat-containing regions were chosen for molecular analysis within this study. Chromosomal DNA was amplified using primers and protocols previously described by Gomes *et al.* [6]. All primers utilized in this study were synthesized by Integrated DNA Technologies, Inc. (Coralville, Iowa, USA). For PCR amplification, approximately 20–30 ng of template DNA was added to a 100 µL reaction containing 0.02 U/µL of Platinum® Taq DNA polymerase High Fidelity (Invitrogen Corporation, Carlsbad, California, USA), 1X PCR buffer (60 mM Tris-SO_4_ (pH 8.0), 18 mM ammonium sulfate), 2 mM MgSO_4_, 0.3 mM dNTPs, 0.3 µM of each primer, and 2% (v/v) dimethyl sulfoxide. PCR was conducted using an iCyclerTM thermal cycler (Bio-Rad Laboratories, Hercules, California, USA) with the following cycling parameters: 1 cycle of 5 min. at 95°C; 40 cycles of 30 sec. at 94°C, 60 sec. at annealing temperature [6], [10], 60 sec. at 72°C; 1 cycle of 10 min. at 72°C; hold at 4°C.

### DNA sequencing

Following DNA amplification, PCR products were purified using isopropanol precipitation and subjected to Sanger sequencing [16] at The Florida State University DNA Sequencing Facility (Tallahassee, Florida, USA). Forward and reverse reads were generated for all amplicons and analyzed using BioEdit Sequence Alignment Editor [17] and MEGA 4.1 [18]. While the use of these sequence analysis programs was sufficient for the *fnb* genes, additional DNA analysis tools were developed for the *clf* genes.

### Sequence analysis of *clf* genes

To analyze the highly variable serine-aspartic acid (SD) repeat region of *clfA* and *clfB*, we developed sequence analysis software following that described by Koreen *et al.* [7]. Briefly, the program analyzes a series of either *clfA* or *clfB* DNA sequences, beginning SD repeat profiling at the TCN-GAY (where N is any nucleotide and Y is either of the pyrimidines) in the first occurrence of GAT-TCN-GAY. The program then analyzes tandemly repeating blocks of 18 nucleotides (one repeat unit) unless nucleotides 13 to 15 are of the sequence TCN, in which case DNA strand-slippage had occurred and the previous 12 nucleotides are considered as one shortened repeat. Each unique repeat unit is then assigned a number, effectively converting the DNA sequence into a numeric profile. As described in [7], the analysis of *clfB* was terminated with the nucleotide immediately prior to the first occurrence of TCN-GAT-TCA-AGA. For *clfA*, this is the first time such a program has been used in profiling the SD repeats, hence no prior termination sequence has previously been reported. Clumping factor A does not contain the same terminating sequence as *clfB*, and therefore, the program was modified to terminate the analysis with the nucleotide immediately prior to the first occurrence of TCN-AAC-AAT-AAT. Refer to supplemental Text files S1 and S2 for the source code to both, *clfA* and *clfB* (respectively), SD repeat profiling programs.

Using this program, genes of interest were converted to a numeric profile based on the nucleotide sequence of each repeating unit, as well as the order and number of repeats. Therefore, two samples that share identical repeat profiles also share 100% nucleotide identity. The program assigns numbers to unique repeat sequences as they are encountered throughout the dataset, and as such, no inference can be made as to the percent nucleotide similarity between two different repeat numbers (e.g. repeat number one is not necessarily more similar to repeat number two than any other repeat number).

Following the generation of numeric *clf* repeat profiles, an additional program was generated to transform the numeric outputs to color-coded representations. As input, the plotting software uses the numeric *clf* repeat profiles along with a file containing hexadecimal color codes. All repeat units were assigned a uniquely colored box; therefore, all like-colored boxes are 100% identical in nucleotide sequence. Refer to supplemental Text file S3 for the source code to the graphing software utilized in this study.

### Phylogenetic reconstruction of MLST data

To determine the genetic relationship between nasal carriage isolates and those isolates of clinical origin, phylogenetic analyses of the concatenated MLST data were carried out for all isolates analyzed in this study using the Metropolis-Hastings coupled Markov chain Monte Carlo method (BI) in MrBayes v3.1.2 [19], [20]. The concatenated MLST dataset was partitioned by locus with the nucleotide substitution model for each being determined using the Akaike Information Criterion (AIC) within jModelTest v0.1.1 [21], [22]. For loci *arcC, glpF, pta,* and *yqiL* the K80 substitution model [23] was used. For locus *gmk*, the K80 plus Gamma substitution model was employed where Gamma indicates that, in addition to the substitution matrix determined by the model for specific nucleotide pairs, a gamma distribution was also applied to determine the overall substitution rate at each nucleotide site [24]. For loci *aroE* and *tpi*, the SYM [25] plus Gamma substitution model was used in BI runs. Two independent BI runs were carried out using random starting trees with one cold chain and three heated chains. Each run consisted of 5 million generations with every 100 steps being sampled. As verified using Tracer v1.5 [26], stationarity was reached after 500 000 generations and a conservative burn-in of 1.25 million (25%) generations was performed.

### Computational analyses of MLST data

Sequence types were assigned to groups using the eBURST v3 program [27] where all members of a group share six of seven identical loci with at least one other member of the group. Using eBURST to compare nasal carriage strains to clinical isolates, nasal carriage strains were treated as the reference set while clinical isolates were treated as the query set. To identify further relationships between isolates, a minimal spanning network of MLST data was generated using TCS v2.1 [28].

### Statistical analyses of gene variability and evolution

Nucleotide diversities were determined for aligned DNA sequences using DnaSP v5 [29]. Molecular evolutionary analyses, including codon-based Z-tests and dN/dS ratios, were conducted using MEGA 4.1 [18] under the Nei-Gojobori P-distance method [30] using 1 000 bootstrap replicates. Indices of discrimination were calculated for all loci using the Discriminatory Power Calculator (http://biophp.org/stats/discriminatory_power/demo.php), which is a modification of the Simpson's index of discrimination test [31], [32].

## Results

### Multilocus sequence typing reveals genetic associations between nasal carriage and clinical isolates

Multilocus sequence typing (MLST) of all 93 *S. aureus* strains analyzed in this study identified 34 different sequence types (STs). Among the 66 nasal carriage isolates, 26 different STs were observed, four of which were new. Additionally, three new alleles were also identified by this study, all at locus *tpi*.

**Figure 1:**
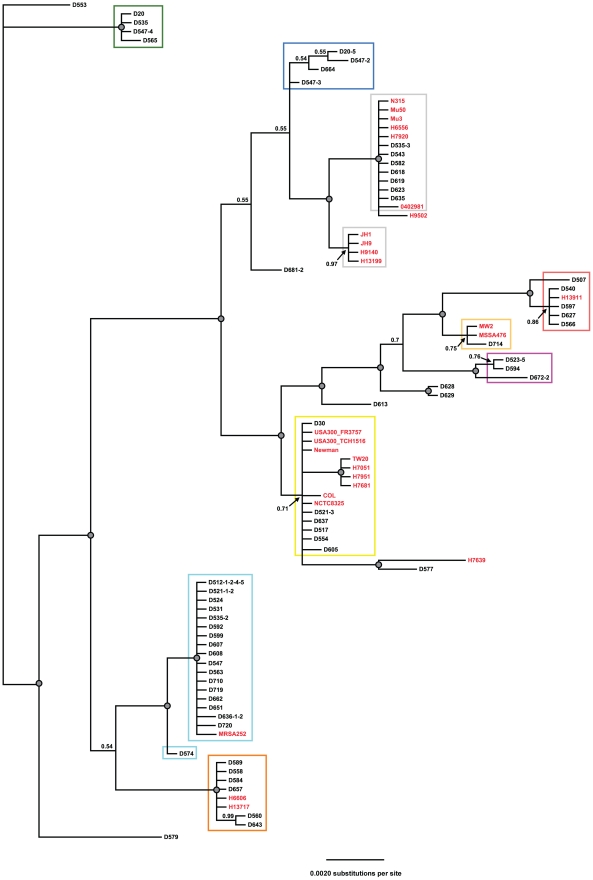
MLST analysis reveals phylogenetic relationships between *S. aureus* nasal carriage and clinical strains. Bayesian phylogram indicating the evolutionary relationships of *S. aureus* strains analyzed in this study. Represented are 66 nasal carriage strains (strains colored in black) and 27 clinical strains (strains colored in red). Note that several clinical isolates cluster on the same genetic clade as major clinical strains. Numbers represent posterior probabilities and grey-filled circles represent nodes receiving 100% posterior probability support. Colored boxes are consistent with strain groupings in Figure 2 (below).

Within the cohort of nasal carriage strains analyzed herein, ST30 was most prevalent, accounting for ∼29% of all isolates. Sequence types 5 and 8 were also prevalent among the nasal carriage strains analyzed within this study, together accounting for ∼20% of all isolates tested (Table S1). While none of the clinical isolates analyzed in this study are of ST30, a combined ∼32% belong to ST5 and ST8. The observation of nasal carriage and clinical isolates belonging to ST5 and ST8 is in agreement with previous reports in which both nasal carriage and clinical isolates belong to these same major clusters [9], [15], [27]. Interestingly, at the ST level, over half of the clinical isolates analyzed in this study (∼54%) belonged to STs (such as ST105 and ST239) that do not contain nasal carriage strains (Table S1). However, phylogenetic analyses of concatenated STs of all strains in this study revealed a close relationship among both nasal carriage and clinical isolates of *S. aureus* (Figure 1). As can be seen in Figure 1, the vast majority of clades containing clinical isolates (strain names in red text) also contain nasal carriage strains from the cohort analyzed in this study (strain names in black text).

**Figure 2:**
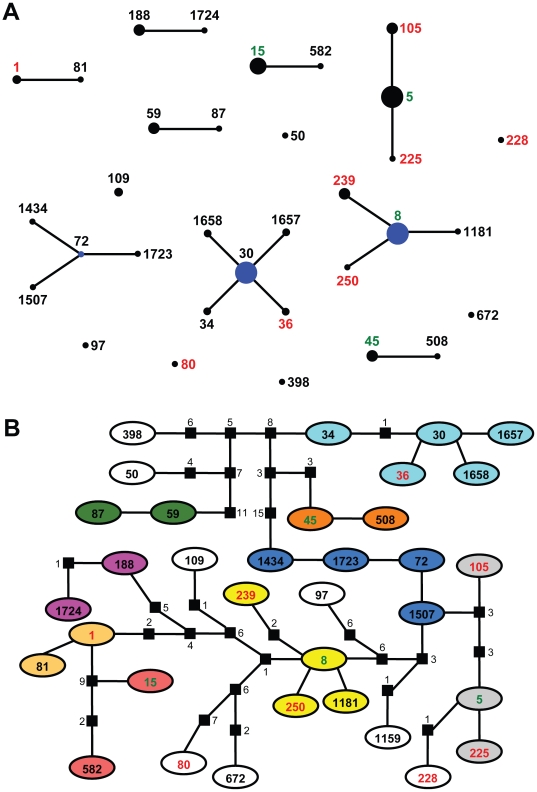
Nasal carriage and clinical isolates of *S. aureus* belong to the same genetic clusters. STs written in black are nasal carriage strains, those written in red are clinical strains and STs written in green indicate both nasal carriage and clinical isolates are contained within. (A) eBURST application of MLST data from all isolates analyzed in this study. Numbers represent ST. STs that are linked by a line belong to the same cluster. Circle sizes are proportional to the number of strains within the ST. (B) Minimal spanning network of MLST data from the same isolates analyzed in (A). Circles represent STs with the numbers within. Branches represent a single nucleotide change between neighboring STs. Black squares indicate multiple nucleotide changes between adjoining STs with the number of differences indicated by the adjacent numbers. Like-colored circles represent STs belonging to the same cluster, as in (A). Non-colored circles are singletons by eBURST analysis.

In addition to estimating phylogenetic relationships among strains we also utilized eBurst, which grouped the strains into nine clusters and eight singletons (Figure 2A). As with the BI, eBURST identified a high degree of relatedness between nasal carriage and clinical isolates. While only four STs contain both nasal carriage and clinical isolates, six of the major clusters contain both classes of isolates. eBURST groupings were based on strains sharing six of seven identical loci and takes into consideration the possibility for genetic recombination; therefore, the level of nucleotide divergence between two different STs contained within the same genetic cluster cannot be determined. To identify whether large-scale nucleotide differences were present between isolates grouped in the same cluster, but belonging to different STs, a minimal spanning network was generated (Figure 2B). This methodology does not take recombination events into consideration, but analyzes all mutations present between samples. The minimal spanning network revealed that many of the strains clustered by eBURST and BI contained only one or few polymorphisms between one another and may further indicate the clonality of the *S. aureus* genome. Collectively, all three computational approaches employed to identify strain relatedness were highly concordant in revealing the genetic associations between nasal carriage strains belonging to the cohort generated for this study and clinical isolates from around the world.

### Virulence gene typing facilitates sub-sequence type strain resolution

**Figure 3:**
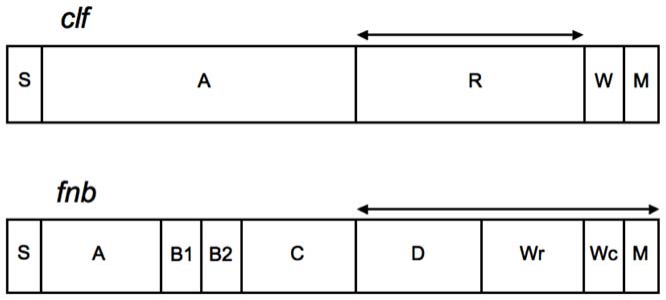
Genetic structure of *clf* and *fnb*. Double-ended arrows indicate region of analysis for this study. For *clf* genes: S, signal sequence; A, fibrinogen/fibronectin-binding domain; R, serine-aspartic acid repeat region; W, wall spanning domain; M, membrane spanning domain. For *fnb* genes: S, signal sequence; A, fibrinogen-binding domain; B, region containing 2 repeats of unknown function (only present in *fnbA*); C, region containing Du repeats with fibronectin-binding activity; D, region containing 4–5 repeats with fibronectin-binding activity; Wr, proline-rich repeat region of the wall spanning domain; Wc, constant region of the wall spanning domain; M, membrane spanning domain.

Since MLST is based on the analysis of slowly evolving housekeeping genes within the *S. aureus* genome, we also analyzed hypervariable virulence-related genes to characterize further the genetic relationships between nasal carriage and clinical isolates. Virulence loci within *clf* and *fnb* gene families were chosen because they have previously been shown to facilitate strain resolution beyond that which is achievable using MLST alone [6], [7]. The genetic diversity of *S. aureus* nasal carriage strains was assessed at *clfA*, *clfB*, *fnbA*, and *fnbB*. Between 52 (*clfA*) and 63 (*fnbA*) different isolates were typed over the hypervariable repeat regions of the *clf* and *fnb* genes (Figure 3), facilitating additional sub-ST strain resolution for 16 out of the 66 (∼24%) nasal carriage isolates that underwent MLST (Table S1). As summarized in Table 1, the *clf* genes were more variable than the *fnb* genes, although both gene families exhibited high levels of genetic diversity, overall. Both *clf* genes exhibited indices of discrimination (ID) of approximately 98.5%. Within the *fnb* loci, both *fnbA* and *fnbB* were highly variable; however, *fnbA* was slightly more variable than *fnbB*. With the exception of *clfB,* increased IDs were observed in this study as compared to those previously reported for epidemic strains of *S. aureus* where the IDs for *clfA*, *fnbA*, and *fnbB* were found to be 87.5%, 62.8%, and 67.9%, respectively [6]. The elevated IDs observed here may, in part, be owed to the larger sample size analyzed within the current study. When epidemic strains were included in the analysis with nasal carriage strains, the IDs for all four genes remained relatively unaffected, albeit slightly lower than with nasal carriage strains alone (Table 1).

**Table 1:** Diversity indices for virulence genes analyzed in this study.

Locus	Regions Analyzed	Includes clinical strains?	# of different strains	# of different haplotypes	Index of Discrimination (ID) (%)	dN/dS ratio
*clfA*	R	Yes	80	54	98.1	0.0750
		No	52	39	98.4	0.0758
*clfB*	R	Yes	89	58	98.4	0.0625
		No	61	44	98.6	0.0641
*fnbA*	D, W, & M	Yes	90	25	89.4	0.2017
		No	63	20	92.1	0.1934
*fnbB*	D, W, & M	Yes	67	22	90.3	0.1498
		No	56	22	91.5	0.1513

### Virulence genes in *S. aureus* provide evidence of purifying selection despite heightened nucleotide diversity

Among the virulence genes analyzed in this study, overall nucleotide diversities were relatively high, with the *clf* genes exhibiting approximately three times more nucleotide diversity than the *fnb* genes (data not shown). Nucleotide diversities represent the average number of nucleotide differences between two sequences at a given site. The nucleotide diversities for *clf* genes were approximately 0.15 while those for the *fnb* genes were approximately 0.05 (data not shown). Despite high nucleotide variability across all loci, strong purifying selection was observed (Table 1) by dN/dS ratios of less than 0.1 at both *clf* loci. Similarly, *fnb* genes exhibited evidence of strong purifying selection (dN/dS ≤ 0.2) despite their heightened nucleotide diversity. These findings suggest that the repeat domains within the virulence genes exhibit a specific and essential function, such that natural selection maintains amino acid homology in spite of high levels of nucleotide substitution.

### Virulence gene repeat domain lengths are identical between nasal carriage and clinical isolates

The R regions of the *clf* genes have previously been assumed to function as a stalk for the extension of the ligand binding domain from the bacterial cell wall [33]. Thus, longer R region lengths may enhance bacterial adherence to nasal epithelia. Since the repeat region of the wall-spanning domain (Wr) within *fnb* genes may also serve a similar function, we extended this hypothesis to include these domains as well. As such, we aimed to elucidate whether longer repeat regions associated with nasal carriage strains as compared to clinical strains of *S. aureus.*

**Figure 4:**
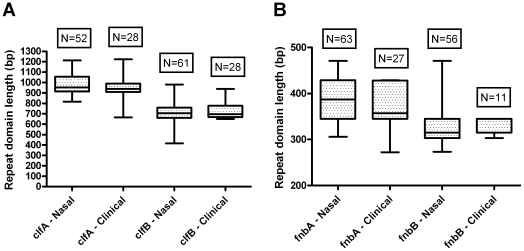
Repeat domain lengths of *clf* and *fnb* genes are indistinguishable between nasal carriage and clinical isolates. Shown are Box and Whisker plots comparing repeat domain lengths between nasal carriage strains of *S. aureus* and clinical strains. (A) Repeat domain lengths compared at *clf* loci; and (B) repeat domain lengths compared at *fnb* loci.

Collectively, a large degree of length variability was observed among both nasal carriage strains of *S. aureus*, as well as clinical strains. For nasal carriage strains, *clf* R region lengths ranged from 816 bp to 1212 bp for *clfA* and 417 bp to 981 bp for *clfB*. Clinical strains analyzed in this study exhibited very similar (and often identical) R region lengths to those of the nasal carrier strains (Figure 4A). Repeat region lengths within clinical strains of *S. aureus* ranged from 666 bp to 1224 bp for *clfA* and 615 bp to 939 bp for *clfB*.

When considering the degrees of DNA strand-slippage at each *clf* locus, slippage events were three to four times more prevalent in *clfA* than *clfB*. Of 178 unique repeats observed in nasal carriage strains at *clfA*, 32 (18%) were 12 nucleotides in length. Of 107 unique repeats observed at *clfB*, only five (4.7%) were 12 nucleotides in length. When all strains analyzed in this study (nasal carriage and clinical) were included in repeat profiling, a total of 185 unique repeats were observed at locus *clfA* with 34 (18.4%) of these being the result of slippage. When all strains were included in the analysis of *clfB*, at total of 109 unique repeats were observed with six (5.5%) being the result of slippage events (refer to supplemental Tables S3 and S4).

When analyzing the nucleotide sequences of the *fnb* genes, the majority of variation laid within the Wr domains in the form of indels, resulting in length differences. No difference was observed in total length of the Wr domains between nasal carriage and clinical isolates (Figure 4B). At *fnbA*, Wr domain lengths ranged from 345 bp to 471 bp for nasal carriage isolates and 273 bp and 429 bp for clinical isolates. At *fnbB*, Wr domain lengths for nasal carriage isolates ranged from 273 bp to 378 bp while clinical isolates ranged from 303 bp and 345 bp. Based on the analysis of repeat domain lengths alone at *clf* and *fnb* loci, it was not possible to distinguish between nasal carriage and clinical strains of *S. aureus*. As such, an analysis of nucleotide sequences was carried out to determine if, at the nucleotide level, nasal carriage and clinical strains belong to the same genetic lineages, or if distinct populations were evident.

### Nasal carriage and clinical isolates of *S. aureus* belong to the same genetic lineages

**Figure 5:**
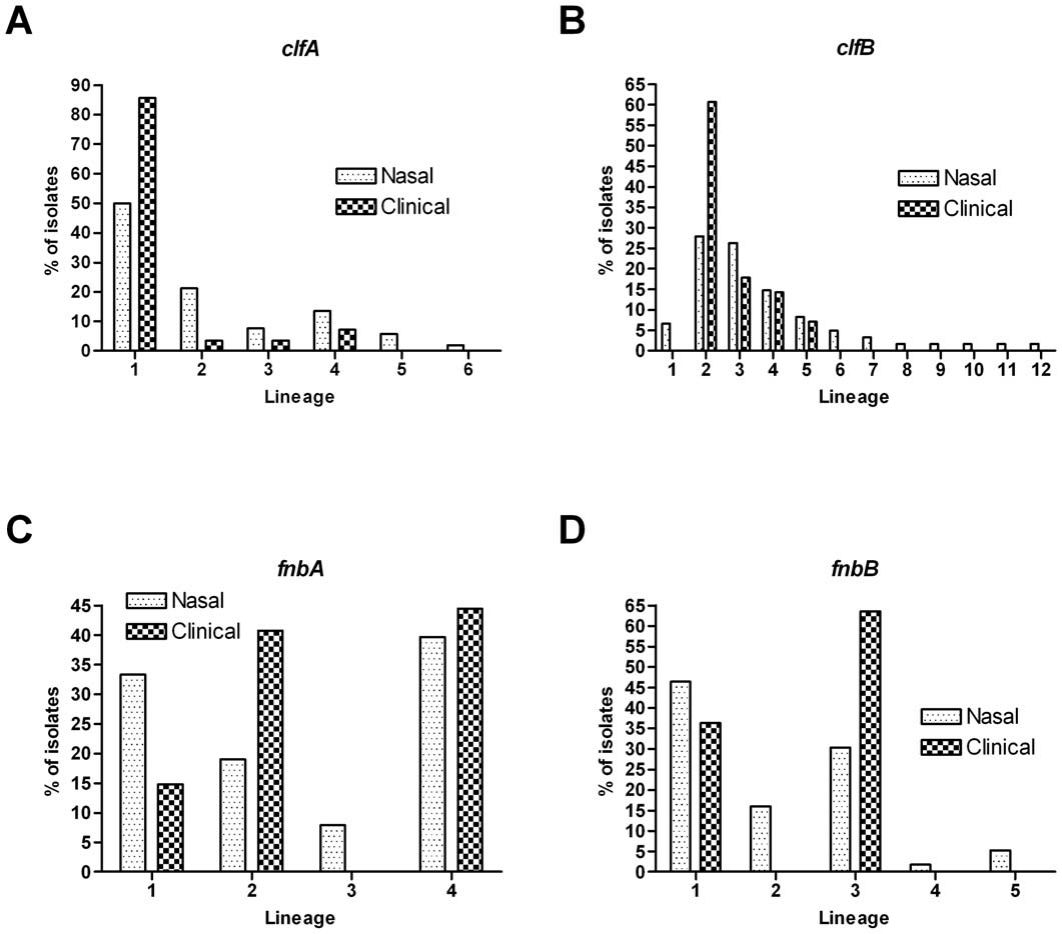
Classification of *S. aureus* strains reveals lineage associations between nasal carriage and clinical isolates. (A) *clfA*, N = 52 nasal carriage and 28 clinical isolates; (B) *clfB*, N = 61 nasal carriage and 28 clinical isolates; (C) *fnbA*, N = 63 nasal carriage and 27 clinical isolates; and (D) *fnbB*, N = 56 nasal carriage and 11 clinical isolates.

To identify relatedness between nasal carriage and clinical isolates of *S. aureus*, *clf* and *fnb* nucleotide sequences were analyzed. Collectively, a large degree of nucleotide sequence diversity among the carrier strains present within our cohort was observed for the *clf* genes, as is indicated by the number of unique repeat units at each locus (refer to supplemental Tables S3 and S4). For *clfA*, 52 nasal carriage isolates were genotyped and 178 unique repeat sequences were identified. Interestingly, *clfB* exhibits a similar index of discrimination to that of *clfA* (Table 1); however, only 107 unique repeat sequences were identified from 61 isolates. While a large degree of variability within the *clf* gene fragments analyzed is the result of point mutations, insertions or deletions of repetitive units are the primary means of variability. As such, strain relatedness cannot be determined using algorithms that rely on sequence alignment [12]. Therefore, in agreement with previous studies [7], [11], [12], [34], [35], [36], lineage assignments were carried out by visual inspection of R domain profiles. On the basis of R domain typing, the 52 nasal carriage isolates for *clfA* were grouped into six lineages (1–6), and 39 haplotypes (Table S1). Lineage 1 was the largest within the sample set containing 26 of 52 (50%) nasal carriage isolates (Figure 5A). Interestingly, 24 of the 28 (85.7%) clinical isolates analyzed herein also belong to this same lineage, including the highly prevalent and virulent USA300 and MW2 (USA400) strains.

When performing the same R domain analysis at locus *clfB*, all 61 nasal carriage strains belonged to 12 lineages (1–12), and 44 different haplotypes (Figure 5B and Table S1). Lineages 2 and 3 contain the most nasal carriage isolates with 17 (27.9%) and 16 (26.2%), respectively. As with *clfA*, a large proportion of the clinical isolates analyzed within this study also belong to these two lineages (including again, USA300). Twenty-two of the 28 (78.6%) clinical isolates analyzed in this study belonged to lineages 2 and 3. Seventeen (60.7%) isolates share lineage 2 with nasal carriage strains while another five (17.9%) belong to lineage 3.

Nucleotide sequence analyses of *S. aureus* isolates were carried out using the D, W (Wr and Wc) and M domains of the *fnb* genes as well. DNA sequence analysis of these domains at locus *fnbA* made it possible to categorize all 63 strains analyzed into four lineages (1–4) while the 56 strains analyzed at *fnbB* were separated into five different lineages (1–5) (Table S1). At both *fnb* loci, two separate lineages were identified that contained the majority of nasal carriage isolates. At *fnbA*, lineages 1 and 4 contained 73% of the nasal isolates with 33.3% and 39.7%, respectively (Figure 5C). At *fnbB*, lineages 1 and 3 were most prevalent within the data set (76.8% of nasal isolates) with 46.4% and 30.4%, respectively (Figure 5D). Within *fnbA*, a total of 59.2% of clinical strains were identified as belonging to lineages 1 and 4 (14.8% and 44.4%, respectively), while 100% of clinical strains, at *fnbB*, belonged to the two most prevalent nasal carriage lineages, 1 and 3 (36.4% and 63.6%, respectively). The lineage assignment data for both *clf* and *fnb* genes identify further the genetic relatedness between the nasal carriage strains analyzed in this study and clinically relevant isolates of *S. aureus.* However, to further verify the relatedness we next sought to determine the prevalence of nasal carriage and clinical strains of *S. aureus* exhibiting identical virulence gene sequences.

### Nasal carriage and clinical isolates of *S. aureus* are identical in *clf* and *fnb* gene sequences

**Figure 6:**
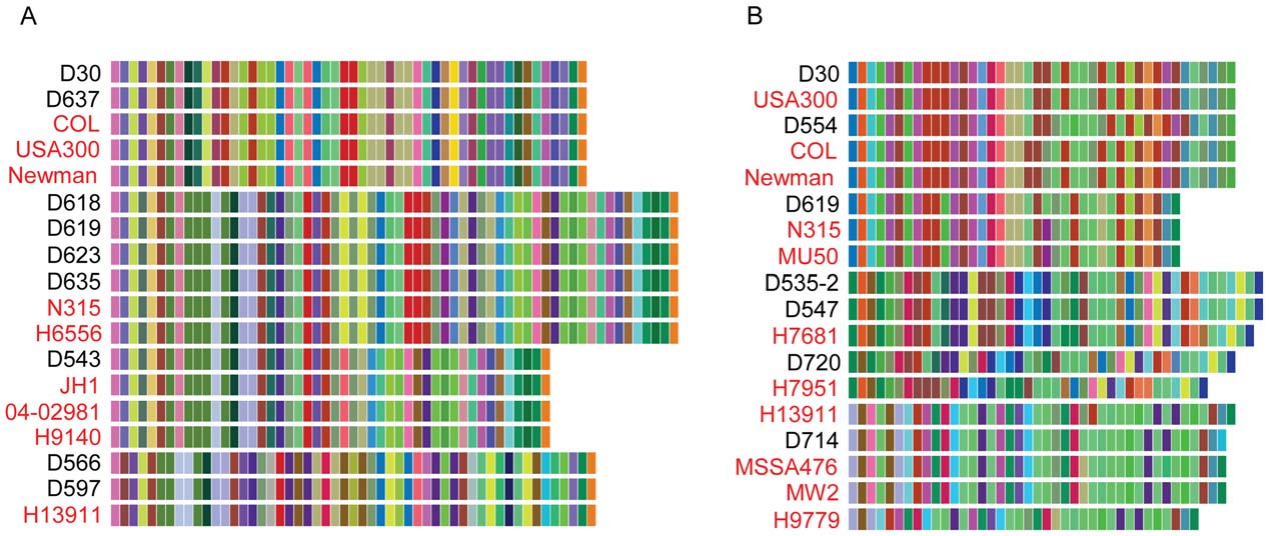
Nasal carriage and clinical isolates of *S. aureus* share (near-)identical *clf* repeat region sequences. Shown are *clf* repeat regions represented as color-coded bars. Like-colored boxes indicate 100% sequence similarity between isolates. (A) *clfA* and (B) *clfB* repeat region sequences from a representative sampling of all isolates analyzed within this study. Isolate names written in black are nasal carriage strains and those written in red are clinical strains.

At locus *clfA*, 11 (39.3%) of the clinical isolates (including USA300) analyzed exhibited identical nucleotide sequence to isolates from healthy donors. Refer to Figure 6A for a representation of the sequence similarities within a subset of the clinical and nasal carriage isolates analyzed herein. For the complete *clfA* data set, refer to supplemental Table S5 and supplemental Figure S1. At *clfB*, nine (32.1%) clinical isolates, including USA300 and MW2, exhibit 100% sequence identity to nasal carrier strains analyzed in this study. Refer to Figure 6B for a comparison of R regions from a subset of nasal carrier and clinical isolates. Refer to supplemental Table S6 and supplemental Figure S2 for the complete *clfB* data set.

**Figure 7:**
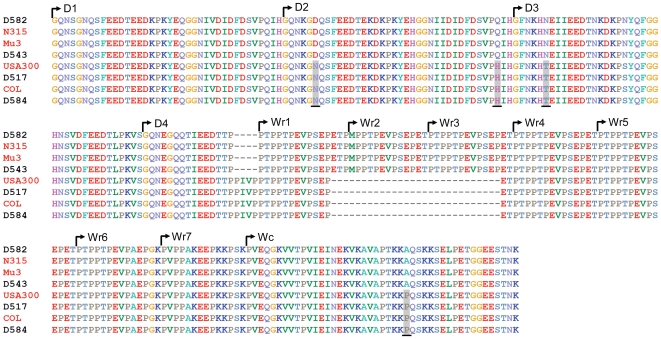
Comparison of FnbA amino acid sequences between four representative nasal carriage and four representative clinical *S. aureus* isolates. Note that identical sequences are evident between both nasal carriage and clinical isolates. D1–D4, repeat region containing fibronectin-binding activity; Wr1–Wr7, proline-rich repeat region of the wall spanning domain; Wc, constant region of the wall spanning domain. Isolate names written in black are nasal carriage strains and those written in red are clinical strains. Regions of amino acid variability are shaded grey and underlined.

When considering the D and W domains of the *fnb* genes, a large percent of clinical isolates exhibited similar, and in many cases identical, genetic sequences to the nasal carriage strains. In fact, 22 (81.5%) of the clinical isolates at locus *fnbA* shared 100% nucleotide sequence identity with nasal carriage isolates. Similarly, for *fnbB*, the same was observed for 8 (72.7%) of the clinical isolates analyzed in this study. Among the clinical strains exhibiting 100% sequence identity with nasal carriage strains at *fnbA* are USA300, N315 and COL (Figure 7). Shown in Figure 7 is an amino acid alignment of eight *S. aureus* strains analyzed in this study (four nasal carriage and four clinical strains) revealing the large degree of homology between the two classifications of strains. At *fnbB,* USA300 was again observed to exhibit 100% sequence identity with nasal carriage strains (data not shown), further supporting the relatedness of nasal carriage strains from the cohort analyzed in this study and clinical isolates of worldwide origin.

## Discussion

Due to the increasing public health concern regarding the severity and rates of *S. aureus* infection throughout the world, it was the goal of this study to perform an evolutionary genetic analysis of nasal carriage strains of *S. aureus* from a healthy population while also analyzing the relatedness of these isolates to strains responsible for pathogenic infection. Using MLST data, computational analyses were conducted to determine the relatedness of clinical and nasal carriage isolates. Bayesian estimation of strain relatedness identified clades containing both nasal carriage and clinical isolates, indicating that these two groups share a recent common ancestor. One drawback to this approach is that, presumably due to the low variability and slow rate of evolution within housekeeping genes, low posterior probabilities are observed due to lack of genetic variability. As such, eBURST and a maximum parsimony minimum spanning network analyses were carried out. eBURST takes into consideration the fact that genetic recombination events within bacterial genomes occur and therefore, highly related strains may still exhibit large-scale nucleotide variation at one or few loci. Here, we generated eBURST clusters requiring six of seven identical loci between strains. Using this method, all of the strains analyzed in this study (nasal carriage and clinical) were clustered into few groups, identifying high genetic relatedness between the nasal carriage strains analyzed in this study and clinical isolates previously identified from around the world.

While many bacterial genomes exhibit high levels of genetic recombination, *S. aureus* has previously been shown to be highly clonal [9], [15], and therefore, a minimal spanning network based on maximum parsimony analysis was also conducted. Once again, the MLST data identified that nasal carriage and clinical strains are genetically related, with only few, if any, single nucleotide polymorphisms present between groups. Moreover, many of the sequence types (STs) that were clustered using BI and eBURST were also identified by this method as being closely related.

Collectively, MLST of nasal carriage and clinical strains in this study indicates that these strains share common genetic lineages based on housekeeping gene fragments in the common minimal genome. This finding unto itself is not surprising given previous reports of nasal carriage and clinical isolates sharing common lineages [9], [15], [27] and that the nature of MLST limits the distinction between strains, which may in turn over represent the relatedness of nasal carriage and clinical isolates. To increase discrimination between strains virulence genes were also analyzed. Not only are virulence genes attractive because of their suitability for analyzing small geographic regions and providing sub-sequence type resolution [6], [11], [12], but also because of their involvement in the pathogenicity of *S. aureus*. Adhesion genes from the clumping factor gene family (specifically, *clfB*) are known to play a significant role in nasal colonization [37] while fibronectin binding protein genes are major contributors to the virulence of *S. aureus* in clinical settings [38]. A number of reports utilized genetic typing of these virulence genes for their discriminatory capabilities between isolates, particularly those belonging to the same ST. By contrast, the current study is, to our knowledge, the first report on the sequence diversity of virulence-related genes from nasal carriage strains of *S. aureus* from a cohort of healthy individuals in the United States.

Here, we have observed a discriminatory power among the virulence genes superior to that reported previously for only clinical strains [6]. This discrepancy may, in part, be owed to the ethnic diversity within our study area, but may also be an indication of the complex evolutionary processes of *S. aureus*; namely the potential for nasal carriage strains to continuously adapt to new environments and evade the host immune system (via continual host-to-host transmission). Using the enhanced discriminatory power of virulence genes, sub-ST strain resolution was achieved for a large percent (∼24%) of strains analyzed in this study. The heightened discriminatory ability of virulence genes was attributable to the high degree of genetic variability at these loci. Interestingly, while a high level of genetic variability was identified among the *clf* and *fnb* loci, a strong indication of purifying selection was still observed. Purifying selection is a reflection that, on average, little to no adaptive diversification (positive selection) of the gene's protein product is being maintained in the population [39], thus suggesting a possible role in the preservation of a specific function.

In order to assess the relatedness of nasal carriage isolates obtained for this study to clinical isolates, the first analysis identified length differences within the repeat regions of the *clf* and *fnb* genes. The repeat regions of the *clf* and *fnb* genes are highly variable in their number of repeating units and these regions are presumed to show the greatest variability between different strains. Discrimination based on the number of repeat units within these regions did not facilitate distinction between nasal carriage strains and clinical isolates. Perhaps the mere presence of determinant virulence genes is sufficient to confer pathogenicity and is not necessarily related to any genetic differences within virulence genes between strains. This hypothesis requires further exploration, as few studies have focused their efforts on the contributions of different genetic regions to pathogen virulence. Initial support to this hypothesis is observed with ClfA. Interestingly, the ClfA protein requires a minimum of 80 amino acid residues within its repeat domain [40]; below this critical length, reduced ClfA activity is observed, while no obvious difference in activity is observed when the length increases. It is possible that the length of the repeat domains within *clfB* or *fnb* genes is sufficient for protein function and as long as they are greater than an, as yet unknown, minimum length, protein function and consequential virulence are indistinguishable between strains.

While differences in repeat length may not directly promote virulence, they may be important in the ability of a strain to colonize nasal epithelia. The difference between persistent, intermittent, or non-carriage may be dependent upon the repeat domain lengths of adhesion genes. Such a consideration has previously been addressed for the coagulase and protein A genes, with no correlation between carriage status and repeat length identified [41]; however, never before has such a longitudinal study been conducted for the *clf* or *fnb* genes, which were previously identified as putative determinants of nasal carriage.

Variable number of tandem repeat profiling did not yield clear distinctions between nasal carriage and clinical isolates, and as such an analysis of nucleotide sequence variability was subsequently carried out for *clf* and *fnb* genes. With increasing frequency, analyses such as this for the *clf* genes are being conducted [6], [7], [42], [43], [44], and we feel that a database, much like that for *spa* typing and MLST, would be beneficial for the transfer and continuity of R domain data between laboratories. The software for R region profiling used in this study is publicly available, and could be combined in a database with all previous repeat sequences and profiles to facilitate faithful identification of strains worldwide. The large amount of data being obtained for *clf* genes and the observed importance of these genes in the classification of strains necessitates the transfer and continuity of genetic data between laboratories. Using the software for *clf* repeat region profiling developed in this study and multiple sequence analysis of *fnb* genes, the classification of nasal carriage and clinical isolates into genetic lineages was conducted.

A high proportion of clinical isolates analyzed in this study (>75%) belonged to the same genetic lineages as nasal carriage strains, revealing an evolutionary relationship stronger than has heretofore been identified. That being said, it is recognized that a full appreciation for the genetic relatedness between nasal carriage and clinical isolates will require future endeavors with employment of large scale molecular typing on large cohorts of both nasal carriage and clinical isolates from the same geographic region. Here we have provided a foundation from which to build upon where a diverse population of clinical strains from around the world has shown genetic relatedness to strains from a nasal carriage population. Furthermore, as next generation sequencing becomes a more feasible option for bacterial typing, and strain collections become more prevalent, the relatedness of nasal carriage and clinical isolates will be more easily and reliably identified.

Of particular interest to this study are the molecular similarities between nasal carriage strains and the highly virulent community-associated methicillin-resistant (CA-MRSA) strains USA300 and MW2 (USA400). These are the two most prevalent strains responsible for CA-MRSA infection within the United States, responsible for 97–99% of all community-acquired skin and soft tissue infections [45], [46], [47]. Interestingly, while USA300 and MW2 do not belong to the same ST, they do share common lineages to each other at *clfA* and *fnbB* (Table S1). They also share common lineages with many nasal carriage strains at all four virulence genes analyzed, belonging to the same lineages as the majority of nasal carriage strains in all cases. Collectively, nucleotide diversities within the repeat regions of *clf* and *fnb* loci facilitate high discriminatory power between strains; however, the molecular data do not distinguish the nasal carriage strains belonging to the cohort analyzed in this study from clinical isolates. In fact, the molecular population analyses indicate that nasal carrier strains share molecular lineages, and are often genetically identical to those strains of clinical significance. While the virulence of a bacterial strain is undoubtedly multifactorial, involving an as yet unknown number of virulence genes, not to mention host factors, the genetic associations identified within this study between clinical and nasal carriage isolates suggest that strain relatedness between nasal carriage and clinical isolates may be higher than has previously been recognized.

## Supporting Information

Figure S1 Color-coded repeats of *clfA* R domains. Shown are color-coded *clfA* R domain profiles for all *S. aureus* strains analyzed in this study.
(TIF)

Figure S2 Color-coded repeats of *clfB* R domains. Shown are color-coded *clfB* R domain profiles for all *S. aureus* strains analyzed in this study.
(TIF)

Table S1 Genotyping details for S. aureus isolates analyzed in this study.
(PDF)

Table S2 GenBank accession numbers for nucleotide sequences utilized/generated in this study.
(PDF)

Table S3 Nucleotide sequences for SD repeats at *clfA*.
(PDF)

Table S4 Nucleotide sequences of SD repeats at *clfB*.
(PDF)

Table S5 Repeat profiles for *clfA*.
(PDF)

Table S6 Repeat profiles for *clfB*.
(PDF)

Text S1 Repeat profiling program for *clfA*.
(PDF)

Text S2 Repeat profiling program for *clfB*.
(PDF)

Text S3 *clf* color-coded repeat generator.
(PDF)
