# Supplementary figures and images for: Evolutionary Analyses of Staphylococcus aureus Identify Genetic Relationships between Nasal Carriage and Clinical Isolates
Source: PLoS One. 2011 Jan 21;6(1):e16426. doi: 10.1371/journal.pone.0016426 (PMC3025037; doi:10.1371/journal.pone.0016426)

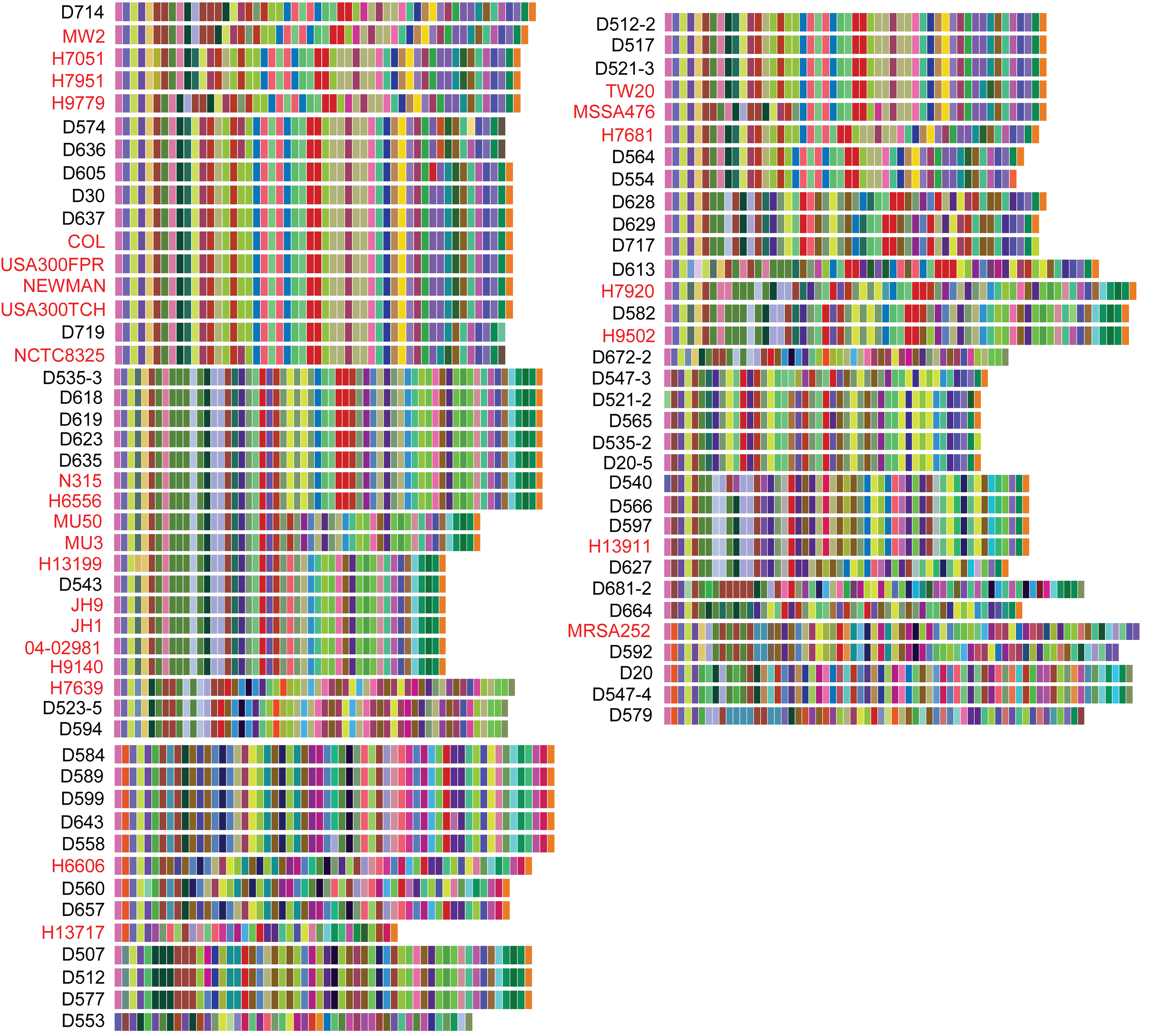

Supplement: Figure S1 — Color-coded repeats of clfA R domains. Shown are color-coded clfA R domain profiles for all S. aureus strains analyzed in this study. (TIF) [file pone.0016426.s001.tif]

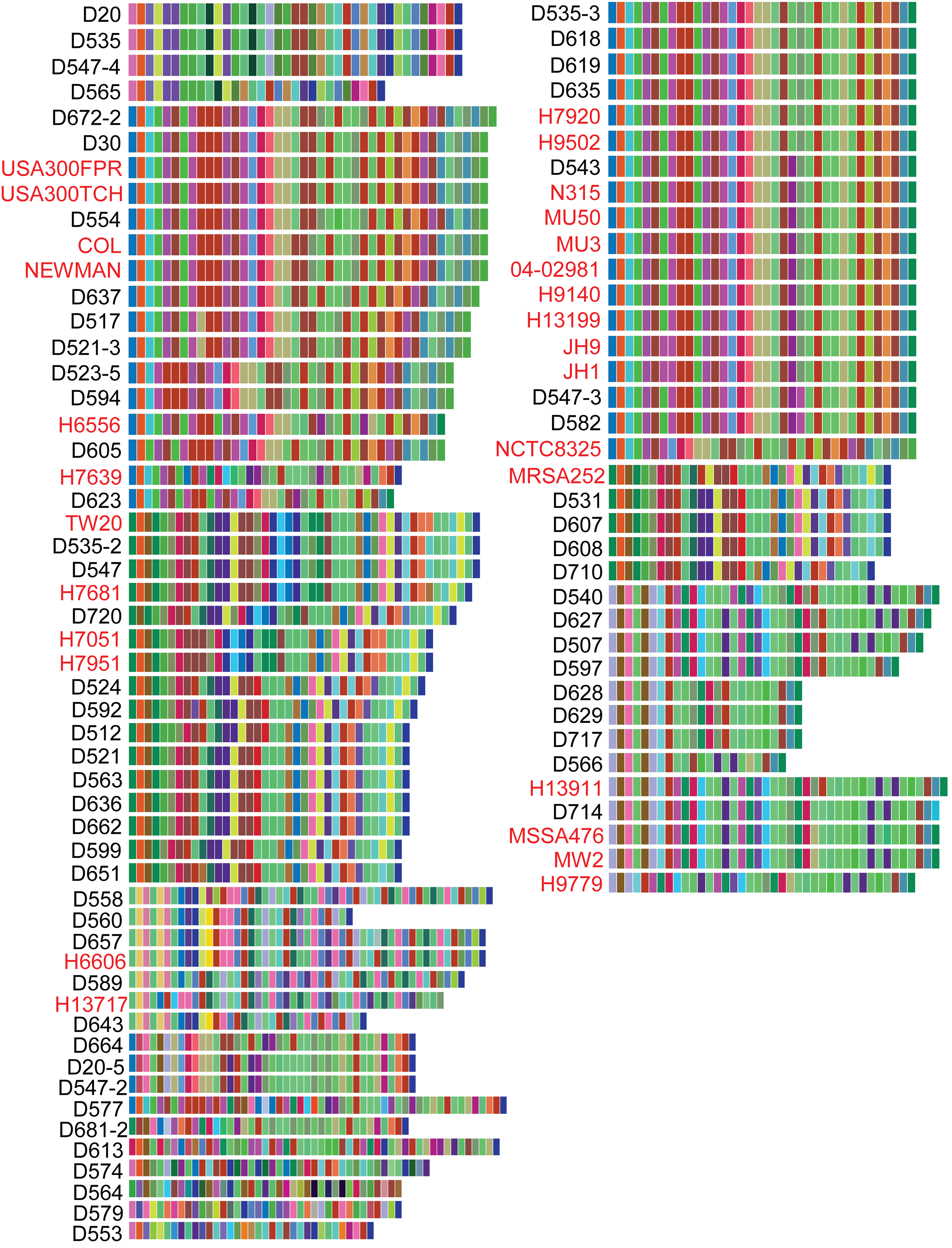

Supplement: Figure S2 — Color-coded repeats of clfB R domains. Shown are color-coded clfB R domain profiles for all S. aureus strains analyzed in this study. (TIF) [file pone.0016426.s002.tif]
