# Supplementary material for: Evolutionary Analyses of Staphylococcus aureus Identify Genetic Relationships between Nasal Carriage and Clinical Isolates
Source: PLoS One. 2011 Jan 21;6(1):e16426. doi: 10.1371/journal.pone.0016426 (PMC3025037; doi:10.1371/journal.pone.0016426)
Supplement: Table S1 — Genotyping details for S. aureus isolates analyzed in this study. (PDF) [file pone.0016426.s003.pdf]

Table S1: Genotyping details for *S. aureus* isolates analyzed in this study.

Sample[a]	Sequence Type (ST)	Genes[b]				Reference
		clfA	*clfB*	*fnbA*	*fnbB*	
MSSA476	1	1	4	1	3	[1]
MW2	1	1	4	1	3	[2]
D535-3	5	1	2	2	1	This study
D543	5	1	2	2	1	This study
D582	5	1	2	2	1	This study
D618	5	1	2	2	1	This study
D619	5	1	2	2	1	This study
D623	5	1	2	2	1	This study
D635	5	1	2	2	1	This study
N315	5	1	2	2	1	[3]
Mu50	5	1	2	2	1	[3]
Mu3	5	1	2	2	1	[4]
H6556	5	1	2	2	NA	[5]
H7920	5	1	2	2	NA	[5]
D30	8	1	2	4	3	This study
D517	8	1	2	4	3	This study
D521-3	8	1	2	4	3	This study
D554	8	1	2	4	3	This study
D637	8	1	2	4	3	This study
USA300_FR3757	8	1	2	4	3	[6]
NCTC8325	8	1	2	4	3	[7]
Newman	8	1	2	NA	NA	[8]
USA300_TCH1516	8	1	2	4	3	[9]
D540	15	2	4	4	3	This study
D566	15	2	4	4	3	This study
D597	15	2	4	4	3	This study
D627	15	2	4	4	3	This study
H13911	15	2	4	4	NA	[5]
D512	30	5	3	4	1	This study
D512-2	30	1	3	4	1	This study
D512-4	30	1	3	4	1	This study
D512-5	30	1	3	1	1	This study
D521	30	NA	3	1	1	This study
D521-2	30	2	3	1	1	This study
D524	30	NA	3	1	1	This study
D531	30	NA	3	NA	1	This study
D535-2	30	2	3	1	1	This study
D547	30	NA	3	1	1	This study
D563	30	NA	3	4	1	This study
D592	30	3	3	1	1	This study
D599	30	4	3	NA	1	This study
D607	30	NA	3	2	1	This study
D608	30	NA	3	1	1	This study
D651	30	NA	3	1	1	This study
D662	30	NA	3	2	1	This study
D710	30	NA	3	2	1	This study
D719	30	1	NA	4	3	This study
D574	34	1	9	4	1	This study
MRSA252	36	3	3	1	NA	[1]
D558	45	4	5	4	2	This study
D584	45	4	NA	4	2	This study
D589	45	4	5	4	2	This study

D657	45	4	5	4	2	This study
H6606	45	4	5	4	NA	[5]
H13717	45	4	5	4	NA	[5]
D553	50	6	12	NA	NA	This study
D20	59	3	1	1	2	This study
D535	59	NA	1	1	2	This study
D547-4	59	3	1	2	2	This study
D664	72	2	6	1	3	This study
H7639	80	1	2	4	NA	[5]
D714	81	1	4	1	3	This study
D565	87	2	1	1	2	This study
D613	97	1	8	1	3	This study
JH9	105	1	2	2	NA	Copeland 2007 Unpub.
JH1	105	1	2	2	NA	Copeland 2007 Unpub.
H9140	105	1	2	2	NA	[5]
H13199	105	1	2	2	NA	[5]
D628	109	1	4	4	NA	This study
D629	109	1	4	3	NA	This study
D523-5	188	1	2	1	5	This study
D594	188	1	2	1	5	This study
04-02981	225	1	2	2	1	[10]
H9502	228	1	2	2	NA	[5]
TW20	239	1	3	4	3	[11]
H7051	239	1	3	4	NA	[5]
H7951	239	1	3	4	NA	[5]
H7681	239	1	3	4	NA	[5]
COL	250	1	2	4	3	[12]
D579	398	3	11	1	1	This study
D560	508	4	5	4	2	This study
D643	508	4	5	4	1	This study
D507	582	5	4	4	3	This study
D577	672	5	7	3	3	This study
D681-2	1159	2	7	3	4	This study
D605	1181	1	2	4	3	This study
D547-2	1434	NA	6	1	1	This study
D547-3	1507	2	2	1	1	This study
D720	1657	NA	3	1	1	This study
D636	1658	1	3	4	1	This study
D636-2	1658	NA	3	2	1	This study
D20-5	1723	2	6	1	3	This study
D672-2	1724	1	2	3	5	This study
D564	NR	1	10	4	NA	This study
D717	NR	1	4	3	NA	This study
H9779	NR	1	4	1	NA	[5]

[a]Sample names beginning in “D” are nasal carriage strains while all others are clinical strains

[b]Number indicates lineage

NR; not reported

NA; no sequence obtained

References:

1. Holden MT, Feil EJ, Lindsay JA, Peacock SJ, Day NP, et al. (2004) Complete genomes of two clinical Staphylococcus aureus strains: evidence for the rapid evolution of virulence and drug resistance. Proc Natl Acad Sci U S A 101: 9786-9791.
2. Baba T, Takeuchi F, Kuroda M, Yuzawa H, Aoki K, et al. (2002) Genome and virulence determinants of high virulence community-acquired MRSA. Lancet 359: 1819-1827.
3. Kuroda M, Ohta T, Uchiyama I, Baba T, Yuzawa H, et al. (2001) Whole genome sequencing of meticillin-resistant Staphylococcus aureus. Lancet 357: 1225-1240.
4. Neoh HM, Cui L, Yuzawa H, Takeuchi F, Matsuo M, et al. (2008) Mutated response regulator graR is responsible for phenotypic conversion of Staphylococcus aureus from heterogeneous vancomycin-intermediate resistance to vancomycin-intermediate resistance. Antimicrob Agents Chemother 52: 45-53.
5. Kuhn G, Francioli P, Blanc DS (2007) Double-locus sequence typing using clfB and spa, a fast and simple method for epidemiological typing of methicillin-resistant Staphylococcus aureus. J Clin Microbiol 45: 54-62.
6. Diep BA, Gill SR, Chang RF, Phan TH, Chen JH, et al. (2006) Complete genome sequence of USA300, an epidemic clone of community-acquired meticillin-resistant Staphylococcus aureus. Lancet 367: 731-739.
7. Gillaspy AF, Worrell V, Orvis J, Roe BA, Dyer W, et al. (2006) The Staphylococcus aureus NCTC 8325 genome. In: Fischetti VA, Novick R, Ferretti J, Portnoy D, Rood J, editors. Gram positive pathogens. Washington, DC: ASM Press. pp. 381-412.
8. Baba T, Bae T, Schneewind O, Takeuchi F, Hiramatsu K (2008) Genome sequence of Staphylococcus aureus strain Newman and comparative analysis of staphylococcal genomes: polymorphism and evolution of two major pathogenicity islands. J Bacteriol 190: 300-310.
9. Highlander SK, Hulten KG, Qin X, Jiang H, Yerrapragada S, et al. (2007) Subtle genetic changes enhance virulence of methicillin resistant and sensitive Staphylococcus aureus. BMC Microbiol 7: 99.
10. Nubel U, Dordel J, Kurt K, Strommenger B, Westh H, et al. (2010) A timescale for evolution, population expansion, and spatial spread of an emerging clone of methicillin-resistant Staphylococcus aureus. PLoS Pathog 6: e1000855.
11. Holden MT, Lindsay JA, Corton C, Quail MA, Cockfield JD, et al. (2010) Genome sequence of a recently emerged, highly transmissible, multi-antibiotic- and antiseptic-resistant variant of methicillin-resistant Staphylococcus aureus, sequence type 239 (TW). J Bacteriol 192: 888-892.
12. Gill SR, Fouts DE, Archer GL, Mongodin EF, Deboy RT, et al. (2005) Insights on evolution of virulence and resistance from the complete genome analysis of an early methicillin-resistant Staphylococcus aureus strain and a biofilm-producing methicillin-resistant Staphylococcus epidermidis strain. J Bacteriol 187: 2426-2438.
