# Supplementary material for: Evolutionary Analyses of Staphylococcus aureus Identify Genetic Relationships between Nasal Carriage and Clinical Isolates
Source: PLoS One. 2011 Jan 21;6(1):e16426. doi: 10.1371/journal.pone.0016426 (PMC3025037; doi:10.1371/journal.pone.0016426)
Supplement: Table S2 — GenBank accession numbers for nucleotide sequences utilized/generated in this study. (PDF) [file pone.0016426.s004.pdf]

Table S2. GenBank accession numbers for nucleotide sequences utilized/generated in this study.

Isolate	Sequence available	GenBank accession #
N315	Whole genome	NC_002745
Mu50	Whole genome	NC_002758
COL	Whole genome	NC_002951
MRSA252	Whole genome	NC_002952
MSSA476	Whole genome	NC_002953
MW2	Whole genome	NC_003923
USA300_FPR3757	Whole genome	NC_007793
NCTC8325	Whole genome	NC_007795
JH1	Whole genome	NC_009632
JH9	Whole genome	NC_009487
Newman	Whole genome	NC_009641
Mu3	Whole genome	NC_009782
USA300_TCH1516	Whole genome	NC_010079
04-02981	Whole genome	CP001844
TW20	Whole genome	FN433596
H6556	Partial *clfA*, SD repeats	AM406905
H7920	Partial *clfA*, SD repeats	AM406930
H13911	Partial *clfA*, SD repeats	AM406870
H6606	Partial *clfA*, SD repeats	AM406906
H13717	Partial *clfA*, SD repeats	AM406861
H7639	Partial *clfA*, SD repeats	AM406923
H9140	Partial *clfA*, SD repeats	AM406950
H13199	Partial *clfA*, SD repeats	AM406847
H9502	Partial *clfA*, SD repeats	AM406958
H7051	Partial *clfA*, SD repeats	AM406914
H7951	Partial *clfA*, SD repeats	AM406931
H7681	Partial *clfA*, SD repeats	AM406924
H9779	Partial *clfA*, SD repeats	AM406961
H6556	Partial *clfB*, SD repeats	AM407049
H7920	Partial *clfB*, SD repeats	AM407074
H13911	Partial *clfB*, SD repeats	AM407014
H6606	Partial *clfB*, SD repeats	AM407050
H13717	Partial *clfB*, SD repeats	AM407005
H7639	Partial *clfB*, SD repeats	AM407067
H9140	Partial *clfB*, SD repeats	AM407094
H13199	Partial *clfB*, SD repeats	AM406991
H9502	Partial *clfB*, SD repeats	AM407102
H7051	Partial *clfB*, SD repeats	AM407058
H7951	Partial *clfB*, SD repeats	AM407075
H7681	Partial *clfB*, SD repeats	AM407068
H9779	Partial *clfB*, SD repeats	AM407105
H6556	Partial *fnbA*, D and W domains	AM407190

H7920	Partial *fnbA*, D and W domains	AM407215
H13911	Partial *fnbA*, D and W domains	AM407158
H6606	Partial *fnbA*, D and W domains	AM407191
H13717	Partial *fnbA*, D and W domains	AM407149
H7639	Partial *fnbA*, D and W domains	AM407208
H9140	Partial *fnbA*, D and W domains	AM407235
H13199	Partial *fnbA*, D and W domains	AM407135
H9502	Partial *fnbA*, D and W domains	AM407243
H7051	Partial *fnbA*, D and W domains	AM407199
H7951	Partial *fnbA*, D and W domains	AM407216
H7681	Partial *fnbA*, D and W domains	AM407209
H9779	Partial *fnbA*, D and W domains	AM407246
D20	Partial *clfA*, SD repeats	HQ325854
D20-5	Partial *clfA*, SD repeats	HQ325855
D30	Partial *clfA*, SD repeats	HQ325856
D507	Partial *clfA*, SD repeats	HQ325857
D512	Partial *clfA*, SD repeats	HQ325858
D512-2	Partial *clfA*, SD repeats	HQ325859
D517	Partial *clfA*, SD repeats	HQ325860
D521-2	Partial *clfA*, SD repeats	HQ325861
D521-3	Partial *clfA*, SD repeats	HQ325862
D523-5	Partial *clfA*, SD repeats	HQ325863
D535-2	Partial *clfA*, SD repeats	HQ325864
D535-3	Partial *clfA*, SD repeats	HQ325865
D540	Partial *clfA*, SD repeats	HQ325866
D543	Partial *clfA*, SD repeats	HQ325867
D547-3	Partial *clfA*, SD repeats	HQ325868
D547-4	Partial *clfA*, SD repeats	HQ325869
D553	Partial *clfA*, SD repeats	HQ325870
D554	Partial *clfA*, SD repeats	HQ325871
D558	Partial *clfA*, SD repeats	HQ325872
D560	Partial *clfA*, SD repeats	HQ325873
D564	Partial *clfA*, SD repeats	HQ325874
D565	Partial *clfA*, SD repeats	HQ325875
D566	Partial *clfA*, SD repeats	HQ325876
D574	Partial *clfA*, SD repeats	HQ325877
D577	Partial *clfA*, SD repeats	HQ325878
D579	Partial *clfA*, SD repeats	HQ325879
D582	Partial *clfA*, SD repeats	HQ325880
D584	Partial *clfA*, SD repeats	HQ325881
D589	Partial *clfA*, SD repeats	HQ325882
D592	Partial *clfA*, SD repeats	HQ325883
D594	Partial *clfA*, SD repeats	HQ325884
D597	Partial *clfA*, SD repeats	HQ325885
D599	Partial *clfA*, SD repeats	HQ325886

D605	Partial *clfA*, SD repeats	HQ325887
D613	Partial *clfA*, SD repeats	HQ325888
D618	Partial *clfA*, SD repeats	HQ325889
D619	Partial *clfA*, SD repeats	HQ325890
D623	Partial *clfA*, SD repeats	HQ325891
D627	Partial *clfA*, SD repeats	HQ325892
D628	Partial *clfA*, SD repeats	HQ325893
D629	Partial *clfA*, SD repeats	HQ325894
D635	Partial *clfA*, SD repeats	HQ325895
D636	Partial *clfA*, SD repeats	HQ325896
D637	Partial *clfA*, SD repeats	HQ325897
D643	Partial *clfA*, SD repeats	HQ325898
D657	Partial *clfA*, SD repeats	HQ325899
D664	Partial *clfA*, SD repeats	HQ325900
D672-2	Partial *clfA*, SD repeats	HQ325901
D681-2	Partial *clfA*, SD repeats	HQ325902
D714	Partial *clfA*, SD repeats	HQ325903
D717	Partial *clfA*, SD repeats	HQ325904
D719	Partial *clfA*, SD repeats	HQ325905
D20	Partial *clfB*, SD repeats	HQ325906
D20-5	Partial *clfB*, SD repeats	HQ325907
D30	Partial *clfB*, SD repeats	HQ325908
D507	Partial *clfB*, SD repeats	HQ325909
D512	Partial *clfB*, SD repeats	HQ325910
D517	Partial *clfB*, SD repeats	HQ325911
D521	Partial *clfB*, SD repeats	HQ325912
D521-3	Partial *clfB*, SD repeats	HQ325913
D523-5	Partial *clfB*, SD repeats	HQ325914
D524	Partial *clfB*, SD repeats	HQ325915
D531	Partial *clfB*, SD repeats	HQ325916
D535	Partial *clfB*, SD repeats	HQ325917
D535-2	Partial *clfB*, SD repeats	HQ325918
D535-3	Partial *clfB*, SD repeats	HQ325919
D540	Partial *clfB*, SD repeats	HQ325920
D543	Partial *clfB*, SD repeats	HQ325921
D547	Partial *clfB*, SD repeats	HQ325922
D547-2	Partial *clfB*, SD repeats	HQ325923
D547-3	Partial *clfB*, SD repeats	HQ325924
D547-4	Partial *clfB*, SD repeats	HQ325925
D553	Partial *clfB*, SD repeats	HQ325926
D554	Partial *clfB*, SD repeats	HQ325927
D558	Partial *clfB*, SD repeats	HQ325928
D560	Partial *clfB*, SD repeats	HQ325929
D563	Partial *clfB*, SD repeats	HQ325930
D564	Partial *clfB*, SD repeats	HQ325931

D565	Partial *clfB*, SD repeats	HQ325932
D566	Partial *clfB*, SD repeats	HQ325933
D574	Partial *clfB*, SD repeats	HQ325934
D577	Partial *clfB*, SD repeats	HQ325935
D579	Partial *clfB*, SD repeats	HQ325936
D582	Partial *clfB*, SD repeats	HQ325937
D589	Partial *clfB*, SD repeats	HQ325938
D592	Partial *clfB*, SD repeats	HQ325939
D594	Partial *clfB*, SD repeats	HQ325940
D597	Partial *clfB*, SD repeats	HQ325941
D599	Partial *clfB*, SD repeats	HQ325942
D605	Partial *clfB*, SD repeats	HQ325943
D607	Partial *clfB*, SD repeats	HQ325944
D608	Partial *clfB*, SD repeats	HQ325945
D613	Partial *clfB*, SD repeats	HQ325946
D618	Partial *clfB*, SD repeats	HQ325947
D619	Partial *clfB*, SD repeats	HQ325948
D623	Partial *clfB*, SD repeats	HQ325949
D627	Partial *clfB*, SD repeats	HQ325950
D628	Partial *clfB*, SD repeats	HQ325951
D629	Partial *clfB*, SD repeats	HQ325952
D635	Partial *clfB*, SD repeats	HQ325953
D636	Partial *clfB*, SD repeats	HQ325954
D637	Partial *clfB*, SD repeats	HQ325955
D643	Partial *clfB*, SD repeats	HQ325956
D651	Partial *clfB*, SD repeats	HQ325957
D657	Partial *clfB*, SD repeats	HQ325958
D662	Partial *clfB*, SD repeats	HQ325959
D664	Partial *clfB*, SD repeats	HQ325960
D672-2	Partial *clfB*, SD repeats	HQ325961
D681-2	Partial *clfB*, SD repeats	HQ325962
D710	Partial *clfB*, SD repeats	HQ325963
D714	Partial *clfB*, SD repeats	HQ325964
D717	Partial *clfB*, SD repeats	HQ325965
D720	Partial *clfB*, SD repeats	HQ325966
D20	Partial *fnbA*, D and W domains	HQ325967
D523-5	Partial *fnbA*, D and W domains	HQ325968
D521	Partial *fnbA*, D and W domains	HQ325969
D524	Partial *fnbA*, D and W domains	HQ325970
D535	Partial *fnbA*, D and W domains	HQ325971
D565	Partial *fnbA*, D and W domains	HQ325972
D594	Partial *fnbA*, D and W domains	HQ325973
D608	Partial *fnbA*, D and W domains	HQ325974
D535-2	Partial *fnbA*, D and W domains	HQ325975
D547	Partial *fnbA*, D and W domains	HQ325976

D592	Partial *fnbA*, D and W domains	HQ325977
D512-5	Partial *fnbA*, D and W domains	HQ325978
D720	Partial *fnbA*, D and W domains	HQ325979
D579	Partial *fnbA*, D and W domains	HQ325980
D521-2	Partial *fnbA*, D and W domains	HQ325981
D20-5	Partial *fnbA*, D and W domains	HQ325982
D664	Partial *fnbA*, D and W domains	HQ325983
D547-2	Partial *fnbA*, D and W domains	HQ325984
D613	Partial *fnbA*, D and W domains	HQ325985
D651	Partial *fnbA*, D and W domains	HQ325986
D714	Partial *fnbA*, D and W domains	HQ325987
D710	Partial *fnbA*, D and W domains	HQ325988
D582	Partial *fnbA*, D and W domains	HQ325989
D635	Partial *fnbA*, D and W domains	HQ325990
D543	Partial *fnbA*, D and W domains	HQ325991
D636-2	Partial *fnbA*, D and W domains	HQ325992
D618	Partial *fnbA*, D and W domains	HQ325993
D607	Partial *fnbA*, D and W domains	HQ325994
D547-4	Partial *fnbA*, D and W domains	HQ325995
D535-3	Partial *fnbA*, D and W domains	HQ325996
D623	Partial *fnbA*, D and W domains	HQ325997
D662	Partial *fnbA*, D and W domains	HQ325998
D619	Partial *fnbA*, D and W domains	HQ325999
D629	Partial *fnbA*, D and W domains	HQ326000
D717	Partial *fnbA*, D and W domains	HQ326001
D577	Partial *fnbA*, D and W domains	HQ326002
D672-2	Partial *fnbA*, D and W domains	HQ326003
D681-2	Partial *fnbA*, D and W domains	HQ326004
D560	Partial *fnbA*, D and W domains	HQ326005
D657	Partial *fnbA*, D and W domains	HQ326006
D563	Partial *fnbA*, D and W domains	HQ326007
D643	Partial *fnbA*, D and W domains	HQ326008
D558	Partial *fnbA*, D and W domains	HQ326009
D589	Partial *fnbA*, D and W domains	HQ326010
D507	Partial *fnbA*, D and W domains	HQ326011
D540	Partial *fnbA*, D and W domains	HQ326012
D597	Partial *fnbA*, D and W domains	HQ326013
D627	Partial *fnbA*, D and W domains	HQ326014
D512	Partial *fnbA*, D and W domains	HQ326015
D566	Partial *fnbA*, D and W domains	HQ326016
D719	Partial *fnbA*, D and W domains	HQ326017
D512-2	Partial *fnbA*, D and W domains	HQ326018
D30	Partial *fnbA*, D and W domains	HQ326019
D636	Partial *fnbA*, D and W domains	HQ326020
D521-3	Partial *fnbA*, D and W domains	HQ326021

D517	Partial *fnbA*, D and W domains	HQ326022
D584	Partial *fnbA*, D and W domains	HQ326023
D637	Partial *fnbA*, D and W domains	HQ326024
D574	Partial *fnbA*, D and W domains	HQ326025
D628	Partial *fnbA*, D and W domains	HQ326026
D605	Partial *fnbA*, D and W domains	HQ326027
D554	Partial *fnbA*, D and W domains	HQ326028
D564	Partial *fnbA*, D and W domains	HQ326029
D662	Partial *fnbB*, D and W domains	HQ326030
D592	Partial *fnbB*, D and W domains	HQ326031
D531	Partial *fnbB*, D and W domains	HQ326032
D710	Partial *fnbB*, D and W domains	HQ326033
D651	Partial *fnbB*, D and W domains	HQ326034
D599	Partial *fnbB*, D and W domains	HQ326035
D608	Partial *fnbB*, D and W domains	HQ326036
D574	Partial *fnbB*, D and W domains	HQ326037
D636	Partial *fnbB*, D and W domains	HQ326038
D512	Partial *fnbB*, D and W domains	HQ326039
D521	Partial *fnbB*, D and W domains	HQ326040
D607	Partial *fnbB*, D and W domains	HQ326041
D563	Partial *fnbB*, D and W domains	HQ326042
D524	Partial *fnbB*, D and W domains	HQ326043
D720	Partial *fnbB*, D and W domains	HQ326044
D535-2	Partial *fnbB*, D and W domains	HQ326045
D643	Partial *fnbB*, D and W domains	HQ326046
D579	Partial *fnbB*, D and W domains	HQ326047
D618	Partial *fnbB*, D and W domains	HQ326048
D619	Partial *fnbB*, D and W domains	HQ326049
D547-2	Partial *fnbB*, D and W domains	HQ326050
D623	Partial *fnbB*, D and W domains	HQ326051
D582	Partial *fnbB*, D and W domains	HQ326052
D543	Partial *fnbB*, D and W domains	HQ326053
D535-3	Partial *fnbB*, D and W domains	HQ326054
D635	Partial *fnbB*, D and W domains	HQ326055
D20	Partial *fnbB*, D and W domains	HQ326056
D565	Partial *fnbB*, D and W domains	HQ326057
D535	Partial *fnbB*, D and W domains	HQ326058
D547-4	Partial *fnbB*, D and W domains	HQ326059
D657	Partial *fnbB*, D and W domains	HQ326060
D589	Partial *fnbB*, D and W domains	HQ326061
D584	Partial *fnbB*, D and W domains	HQ326062
D558	Partial *fnbB*, D and W domains	HQ326063
D560	Partial *fnbB*, D and W domains	HQ326064
D681-2	Partial *fnbB*, D and W domains	HQ326065
D597	Partial *fnbB*, D and W domains	HQ326066

D613	Partial *fnbB*, D and W domains	HQ326067
D20-5	Partial *fnbB*, D and W domains	HQ326068
D507	Partial *fnbB*, D and W domains	HQ326069
D540	Partial *fnbB*, D and W domains	HQ326070
D664	Partial *fnbB*, D and W domains	HQ326071
D566	Partial *fnbB*, D and W domains	HQ326072
D577	Partial *fnbB*, D and W domains	HQ326073
D627	Partial *fnbB*, D and W domains	HQ326074
D714	Partial *fnbB*, D and W domains	HQ326075
D554	Partial *fnbB*, D and W domains	HQ326076
D719	Partial *fnbB*, D and W domains	HQ326077
D637	Partial *fnbB*, D and W domains	HQ326078
D605	Partial *fnbB*, D and W domains	HQ326079
D521-3	Partial *fnbB*, D and W domains	HQ326080
D30	Partial *fnbB*, D and W domains	HQ326081
D517	Partial *fnbB*, D and W domains	HQ326082
D594	Partial *fnbB*, D and W domains	HQ326083
D523-5	Partial *fnbB*, D and W domains	HQ326084
D672-2	Partial *fnbB*, D and W domains	HQ326085
