# Supplementary material for: Evolutionary Analyses of Staphylococcus aureus Identify Genetic Relationships between Nasal Carriage and Clinical Isolates
Source: PLoS One. 2011 Jan 21;6(1):e16426. doi: 10.1371/journal.pone.0016426 (PMC3025037; doi:10.1371/journal.pone.0016426)
Supplement: Table S3 — Nucleotide sequences for SD repeats at clfA. (PDF) [file pone.0016426.s005.pdf]

Table S3. Nucleotide sequences for SD repeats at *clfA*

Repeat numbers and sequences		Repeat numbers and sequences	
1	TCAGATTCTGACCCAGGT	94	TCAGATTCCGACAGCGAT
2	TCAGATAGTGGT	95	TCAGACAGCGAT
3	TCAGATTCTGGCAGCGAT	96	TCTGACTCAGATAGTGAC
4	TCTAATTCAGATAGCGGT	97	TCCGACTTAGACAGCGAC
5	TCAGATTCGGGTAGTGAT	98	TCCGAGTCAGAT
6	TCTACATCAGATAGTGAT	99	TCAGATTCTGGCAGTGAT
7	TCAGATTCAGATAGTGAT	100	TCAGATTCAGACCCAGGT
8	TCAGATTCAGCAAGCGAT	101	TCAGACTCAGTGAGCGAT
9	TCAGATTCAGCGAGCGAT	102	TCCTACTCAGATAGCGAC
10	TCAGATTCAGCAAGTGAT	103	TCAGACTCGGATAGCGAT
11	TCAGATTCAGCGAGTGAT	104	TCAGAATCAGATAATGAC
12	TCCGACTCCGACAGTGAC	105	TCTGACTCAGGTAGTGAC
13	TCCGACTCAGATAACGAT	106	TCGGATTCAGATAGCGAA
14	TCTGACTCAGACAGTGAC	107	TTAGATTCAGACAGCGAC
15	TCAGACTCAGATAGCGAT	108	TCAGATTCAGGTAGCGAT
16	TCAGATTCAGAGAGCGAT	109	TCAGATTCAGAC
17	TCGGATTCAGATAGTGAT	110	TCCGATTCTGAC
18	TCTGACTCAGACAGCGAC	111	TCCGATTCAGATAGCGGT
19	TCAGACTCAGACAGCGAC	112	TCCGATTCAGCAAGTGAT
20	TCAGACTCAGACAGTGAT	113	TCAGACTCAGAAAGTGAC
21	TCAGATTCCGACAGTGAT	114	TCAAATTCCGATAGCGAT
22	TTAGACTCAGACAGTGAC	115	TCAGATTCCGAC
23	TTAGACTCAGACAGCGAC	116	TCAGGTAGTGCC
24	TCAGACTCAGACAGTGAC	117	TCCGACTCAGACAGTGAT
25	TCAGATTCCGACAGTGAC	118	TCAGACTCAGGTAGTGCC
26	TCGGATTCCGATAGCGAT	119	TCTGATTCAGATAGTGAC
27	TCCGACTCAGACAGCGAC	120	TCAACGAGTGACAAAGAA
28	TCCGACTCAGACAGCGAT	121	TCAGACAATGAC
29	TCCGACTCAGATAGCGAC	122	TCAATAGCGATTCCGAGT
30	TCAGACTCAGACAGCGAT	123	TCAGACTCAAACAGCGAT
31	TCAGATTCAGACAGTGAT	124	TCAGATTTAGCAAGCGAT
32	TCAGATTCCGATAGCGAT	125	TCCGATTCAGCGAGTGAC
33	TCAGAATCAGATAGCGAC	126	TCAGATTCCAACAGTGAC
34	TCCGACTCAGTTAGCGAT	127	TCAGACTCAGATAATGAC
35	TCAGATTCAGATAGCAAT	128	TCAGATTCATCAAGTGAT
36	TCAGAATCAGATAGTGAT	129	TCAGATTTGGGTAGTGAT
37	TCAGATTCCGACAGCGAC	130	TCCGATTCAGCGAGCGAT
38	TCCGACTCAGGTAGTGAC	131	TCAGACTCAGCGAGCGAT
39	TCCGACTCAGATAGTGAT	132	TCAGATTTAGACAGCGAC
40	TCAGATTCAACGAGTGAT	133	TCAGACTCACGTAGTGAC
41	TCCGATTCTGAT	134	TCCGAGTCAGTT
42	TCAACGAGTGACACAGGA	135	TCAGATTCAGTGAGTGAT
43	TCAGACAACGAC	136	TCAGACTCAGAC
44	TCTGACTCAGAAAGTGAT	137	TCAGAATCGGATAGCGAC

45	TCAAATAGCGAT
46	TCCGACTCAGGT
47	TCAGATAGCGGT
48	TCCGACTCAGCGAGCGAT
49	TCAGACTCAGATAGTGAC
50	TCCGATAGCGAT
51	TCAGATTCAGACAGCGAT
52	TCCGACTCAGATAGCGAT
53	TCAGATTCAGACAACGAT
54	TCTGACTCAGACAGCGAT
55	TCCGACTCAGACAGTGAC
56	TCGGATTCAGACAGCGAT
57	TCGGATTCCGACAGTGAT
58	TCAGATTCCGATAGTGAC
59	TCGGATTCAGCGAGTGAT
60	TCCGATTCATCAAGTGAT
61	TCCGACTCAGAAAGTGAT
62	TCCGAGTCAGGT
63	TCTACATCAGATAGTGGT
64	TCAGACTCAGCGAGTGAT
65	TCCGACTCAGACAATGAC
66	TCGGATTCAGATAGCGAT
67	TCAGATTCAGATAGCGAT
68	TCTGACTCCGACAGTGAT
69	TCGGATTCAGATAGCGAC
70	TCAGACTCGGATAGCGAC
71	TCGGACTCAGATAGCGAT
72	TCAGAATCAGACAGCGAT
73	TCAGATTCAGACAGCGAC
74	TCAGACAGTGAC
75	TCAGATTCAGATAGTGAC
76	TCAGACTCAGGTAGTGAC
77	TCAGATTCAGGCAGCGAT
78	TCTACATCAGATAGCGAT
79	TCTGACTCAGATAGCGAT
80	TCAGATTCAGATAGCGAC
81	TCAGACTCAGATAGCGAC
82	TCAGATTCGGATAGCGAT
83	TCAGATTCAGACAGTGAC
84	TCAGAATCAGATAGTGAC
85	TCCGATTCAGACAGCGAT
86	TCCGATTCAGATAGCGAT
87	TCAGATTCCGAT
88	TCAGACAGTGAT
89	TCAGATTCGGACCCAGGT
90	TCAGATAGCGAT
138	TCAGACAGCGAC
139	TCAGAATCAGAAAGCGAC
140	TCCGATTCAGACAGTGAC
141	TCCGACTCAGACAGTGCC
142	TCGGATTCAACGAGTGAC
143	ACAGGATCAGACAACGAC
144	TCTGAGTCAGGT
145	TCAGACTCAGGTAGTGGC
146	TCCGATTCAGCAAGCGAT
147	TCAGACTCAGAAAGCGAC
148	TCAGACAGTGTT
149	TCAGACTCGGATAGTGAA
150	TCCGACTCGGATAGCGAT
151	TCGGATTCCGACAGCGAT
152	TCCGACTCAGATAGTGCC
153	TCCGATTCAGAT
154	TCAGATAACGAC
155	TCAGACTCAGAAAGTGAT
156	TCGAATAGCGAT
157	TCCGATTCAGGT
158	TCAGATTCGGGTAGAGGT
159	TCAGACTCTGGCAGCGAT
160	TCAGACTCAGAT
161	TCTGACTCAGAT
162	TCTGACTCAGACAGTGAT
163	GCAGACTCAGACAGTGAC
164	TCAGATTCACGTAGCGAT
165	TCCGACTCAGATAGTGAC
166	TCCGACTCAGCAAGTGAT
167	TCTAATTCAGATAGCGGC
168	TCAGACTCAGCAAGCGAT
169	TCTGACTCAGAC
170	TCAGACTCAGGTAGTGAT
171	TCCGACTCAGGTAGTGAT
172	TCCGACTCAGGTAGTGCC
173	TCGGATTCAACCAGTGAC
174	ACAGGATCAGATAACGAC
175	TCAGATTCTGACAGTGCC
176	TCGGAATCAGCGAGTGAT
177	TCAGATTCTGAT
178	TCGGAGTCAGGT
179	TCCGACTCGGATAGCGAC
180	TCAGATTCCAATAGCGAT
181	TCAGATTCAGCGAGTGGT
182	TCTACATCAGATAGCGAC
183	TCGGATTCCGAC

91	TCAGATTCGGGTAGTGAC	184	TCAGACTCAGATAACGAT
92	TCAGACTCAGCAAGTGAT	185	TCAAATTCTGGCAGTGAT
93	TCGGATTCAGATAGTGAC		
