# Supplementary material for: Evolutionary Analyses of Staphylococcus aureus Identify Genetic Relationships between Nasal Carriage and Clinical Isolates
Source: PLoS One. 2011 Jan 21;6(1):e16426. doi: 10.1371/journal.pone.0016426 (PMC3025037; doi:10.1371/journal.pone.0016426)
Supplement: Table S4 — Nucleotide sequences of SD repeats at clfB. (PDF) [file pone.0016426.s006.pdf]

Table S4. Nucleotide sequences of SD repeats at *clfB*

| Repeat numbers and sequences | | Repeat numbers and sequences | |
|---|---|---|---|
| 1 | TCGGATTCGGACAGTGAC | 56 | TCAGGTTCAGACAGTGAG |
| 2 | TCAGGCTCAGACAGCGAC | 57 | TCGGACTCAGATAGCAAC |
| 3 | TCAGGTTCAGACAGTGAC | 58 | TCGGATTCGGACAGCGAC |
| 4 | TCGGACTCAGACAGCGAC | 59 | ACAGATTCAGATAGTGAC |
| 5 | TCAGATTCAGATAGTGAC | 60 | ACAGATTCAGACAGCGAC |
| 6 | TCAGACTCAGATAGTGAC | 61 | TCTGATTCAGACAGCGAC |
| 7 | TCAGATTCAGACAGCGAT | 62 | TCCGATTCAGATAGTGAT |
| 8 | TCGGATTTAGACAGCGAT | 63 | TCAGACTCAGGTAGCGAT |
| 9 | TCGGATTCAGACAGCGAC | 64 | TCAGACTCAGATAGTGAG |
| 10 | TCAGATTCAGATAGTGAT | 65 | TCAGATTCCGATAGTGAC |
| 11 | TCAGATTCAGACAGCGAC | 66 | TCCGACTCCGAC |
| 12 | TCAGACTCAGATAGTGAT | 67 | TCCGACAGCGAT |
| 13 | TCAGACTCAGACAGTGAG | 68 | TCCGATTCAGACAGCGAT |
| 14 | TCAGATTCAGATAGCGAT | 69 | TCCGACTCCGACAGCGAT |
| 15 | TCAGACTCAGACAGTGAC | 70 | TCAGATTCAGACAGCGAG |
| 16 | TCCGATTCAGATAGCGAT | 71 | TCCGACACGGACAGCGAC |
| 17 | TCGGACTCAGATAGCGAC | 72 | TCAGATTCAGAAAGTGAC |
| 18 | TCCGATTCAGATAGCGAG | 73 | TCTGATTCAGACAGCGAT |
| 19 | TCAGACTCAGACAGTGAT | 74 | TCAGATTCAGAGAGCGAT |
| 20 | TCGGATTCAGACAGCGAT | 75 | TCCGACTCAGACAGCGAC |
| 21 | TCGGATTCAGACAGTGAC | 76 | TCCGGTTCAGATAGTGAT |
| 22 | TCAGAATCAGACAGTGAT | 77 | TCAGATTCCGACAGCGAT |
| 23 | TCAGACTCAGACAGCGAC | 78 | TCGGATTCCGACAGCGAC |
| 24 | TCAGGTTCAGATAGCGAT | 79 | TCAGATTCCGACAGTGAT |
| 25 | TCAGACTCAGATAGCGAT | 80 | TCCGACTCAGACAGCGAT |
| 26 | TCAGAATCAGATAGTGAG | 81 | TCAGATTCCGACAGCGAC |
| 27 | TCAGATTCAGACAGTGAC | 82 | TCCGATTCAGATAATGAC |
| 28 | TCGGACTCAGACAGTGAT | 83 | TCCGATTCTGATAGTGAC |
| 29 | TCAGACTCAGACAGCGAT | 84 | TCCGACTCTGATAGTGAC |
| 30 | TCAGATTCAGATAGCGAC | 85 | TCTGATTCAGATAGTGAT |
| 31 | TCAGAATCAGACAGCGAC | 86 | TCCGATTCAGACAGTGAC |
| 32 | TCAGACTCAGATAGCGAC | 87 | TCAGACTCAGAAAGCGAT |
| 33 | TCAGAATCAGACAGTGAC | 88 | TCGGACTCAGATAGTGAT |
| 34 | TCAGGTTCAGATAGCGAC | 89 | TCGGATTCAGACAGTGAG |
| 35 | TCAGAATCAGATAGCGAT | 90 | TCCGATTCAGATAGTGAC |
| 36 | TCGGATTCAGACAGTGAT | 91 | TCCGATTCAGACAGTGAG |
| 37 | TCAGAATCAGATAGCGAC | 92 | TCAGGCTCAGACAGCGAT |
| 38 | TCGGACTCAGACAGCGAT | 93 | TCGGATTCAGACAAAGAT |
| 39 | TCAGACTCGGATAGCGAT | 94 | TCAGACTCAGAC |
| 40 | TCAGACTCGGATAGCGAC | 95 | TCAGATAGCGAT |
| 41 | TCGGATTCAGATAGCGAC | 96 | TCAGGCTCAGACAGTGAC |
| 42 | TCAGAATCAGACAGTGAG | 97 | TCAGACTCAGAGAGTGAC |
| 43 | TCAGATTCAGATAGTGAG | 98 | TCAGATTCGGACAGTGAC |
| 44 | TCGGACTCAGATAGCGAT | 99 | TCAGACAGTGAC |

| | | | |
|---|---|---|---|
| 45 | TCGGATTCAGATAGTGAC | 100 | TCAGACTTAGACAGTGAC |
| 46 | TCAAACTCAGACAGTGAG | 101 | TCGGACTCAGAGAGTGAC |
| 47 | TCGGACTCAGATAGTGAC | 102 | TCAGATTTAGATAGCGAC |
| 48 | TCGGACTCAGACAGTGAG | 103 | TCAGATTCGGACAGCGAT |
| 49 | TCGGATTCAAACAGCGAT | 104 | TCAGATTCAGATAGCAAC |
| 50 | TCGGACTCAGACAGTGAC | 105 | TTAGATTCAGATAGCGAT |
| 51 | TCAAACTCAGATAGTGAC | 106 | TCGGATTCAGACAACGAT |
| 52 | TCGGATTCAGATAGCGAT | 107 | TCGGAGTCAGAGAGTGAC |
| 53 | TCAGAATCAGACAGCGAT | 108 | TCAGATAGCGAC |
| 54 | TCAGACCCAGACAGTGAG | 109 | TCAGACCCAGATCCGGAT |
| 55 | TCAGATTCAGACAGTGAG | | |
