# Supplementary material for: Evolutionary Analyses of Staphylococcus aureus Identify Genetic Relationships between Nasal Carriage and Clinical Isolates
Source: PLoS One. 2011 Jan 21;6(1):e16426. doi: 10.1371/journal.pone.0016426 (PMC3025037; doi:10.1371/journal.pone.0016426)
Supplement: Table S5 — Repeat profiles for clfA. (PDF) [file pone.0016426.s007.pdf]

Table S5. Repeat profiles for *clfA*

Lineage	Haplotype	Sample	Numeric Profile
1	1	714	1-3-4-5-63-11-11-10-64-8-9-11-11-11-8-65-66-14-32-14-67-67-21-28-68-28-21-52-29-51-51-67-126-25-66-25-25-20-59-17-12-69-70-66-71-72-72-73-75-59-76-60-61-45-62
	40	MW2	1-3-4-5-63-11-11-10-64-8-9-11-11-8-65-66-14-32-14-67-67-21-28-68-28-21-52-29-51-51-67-126-25-66-25-25-20-59-17-12-69-70-66-71-72-72-73-75-59-76-60-61-45-62
	41	H7051	1-3-4-5-63-181-11-10-64-8-48-65-66-14-25-67-14-67-67-21-28-68-28-21-52-29-51-51-67-25-25-66-25-25-20-59-17-12-69-70-66-71-72-72-73-74-75-59-76-60-61-45-62
		H7951	1-3-4-5-63-181-11-10-64-8-48-65-66-14-25-67-14-67-67-21-28-68-28-21-52-29-51-51-67-25-25-66-25-25-20-59-17-12-69-70-66-71-72-72-73-74-75-59-76-60-61-45-62
	42	H9779	1-3-4-5-63-11-10-64-8-9-11-11-8-65-66-14-32-14-67-67-21-28-68-28-21-52-29-51-51-67-126-25-66-25-25-20-59-17-12-69-70-66-71-72-72-73-75-59-76-60-61-45-62
	2	574	1-3-4-5-63-11-10-64-8-48-65-66-14-25-67-14-66-67-21-28-68-28-21-52-29-51-51-67-25-25-66-25-25-20-59-17-12-69-70-66-71-72-132-74-75-59-133-60-61-45-134
	3	636	1-3-4-5-63-11-10-64-8-48-65-66-14-25-67-14-66-67-21-28-68-28-21-52-29-51-51-67-25-25-66-25-25-20-59-17-12-69-70-66-71-72-132-74-75-59-76-60-61-45-134
	4	605	1-3-4-5-63-11-10-64-8-48-65-66-14-25-67-14-67-67-21-28-68-28-21-52-29-51-51-67-25-25-66-25-25-20-59-17-12-69-70-66-71-51-72-73-74-75-59-76-60-61-45-62
	5	30	1-3-4-5-63-11-10-64-8-48-65-66-14-25-67-14-67-67-21-28-68-28-21-52-29-51-51-67-25-25-66-25-25-20-59-17-12-69-70-66-71-72-72-73-74-75-59-76-60-61-45-62
		637	1-3-4-5-63-11-10-64-8-48-65-66-14-25-67-14-67-67-21-28-68-28-21-52-29-51-51-67-25-25-66-25-25-20-59-17-12-69-70-66-71-72-72-73-74-75-59-76-60-61-45-62
		COL	1-3-4-5-63-11-10-64-8-48-65-66-14-25-67-14-67-67-21-28-68-28-21-52-29-51-51-67-25-25-66-25-25-20-59-17-12-69-70-66-71-72-72-73-74-75-59-76-60-61-45-62
		USA300_FPR3757	1-3-4-5-63-11-10-64-8-48-65-66-14-25-67-14-67-67-21-28-68-28-21-52-29-51-51-67-25-25-66-25-25-20-59-17-12-69-70-66-71-72-72-73-74-75-59-76-60-61-45-62
		Newman	1-3-4-5-63-11-10-64-8-48-65-66-14-25-67-14-67-67-21-28-68-28-21-52-29-51-51-67-25-25-66-25-25-20-59-17-12-69-70-66-71-72-72-73-74-75-59-76-60-61-45-62
		USA300_TCH1516	1-3-4-5-63-11-10-64-8-48-65-66-14-25-67-14-67-67-21-28-68-28-21-52-29-51-51-67-25-25-66-25-25-20-59-17-12-69-70-66-71-72-72-73-74-75-59-76-60-61-45-62
	6	719	1-3-4-5-63-11-10-64-8-48-65-66-14-25-67-14-67-67-21-28-68-28-21-52-29-51-51-67-25-25-66-25-25-20-59-17-12-69-70-66-71-72-72-73-75-59-76-60-61-45-178
	43	NCTC8325	1-3-4-5-63-11-10-64-8-48-65-66-14-25-67-14-66-67-21-28-68-28-21-52-29-51-51-67-25-25-66-25-25-20-59-17-12-69-70-66-71-72-73-74-75-59-76-60-61-45-134
	7	512-2	1-3-4-5-63-11-10-64-8-48-65-66-14-25-67-14-67-67-21-28-68-28-21-52-29-51-51-67-25-25-66-25-25-20-59-17-12-69-70-66-71-72-72-73-75-59-76-60-61-45-62
		517	1-3-4-5-63-11-10-64-8-48-65-66-14-25-67-14-67-67-21-28-68-28-21-52-29-51-51-67-25-25-66-25-25-20-59-17-12-69-70-66-71-72-72-73-75-59-76-60-61-45-62
		521-3	1-3-4-5-63-11-10-64-8-48-65-66-14-25-67-14-67-67-21-28-68-28-21-52-29-51-51-67-

			25-25-66-25-25-20-59-17-12-69-70-66-71-72-72-73-75-59-76-60-61-45-62
	44	TW20	1-3-4-5-63-11-10-64-8-48-65-66-14-25-67-14-67-67-21-28-68-28-21-52-29-51-51-67-25-25-66-25-25-20-59-17-12-69-70-66-71-72-73-74-75-59-76-60-61-45-62
	45	MSSA476	1-3-4-5-63-11-11-10-64-8-9-11-11-8-65-66-14-32-21-28-68-28-21-52-29-51-51-7-126-25-66-25-25-20-59-17-12-69-70-66-71-72-72-73-75-59-76-60-61-45-62
	46	H7681	1-3-4-5-63-11-10-64-8-48-65-66-14-25-67-14-67-67-21-28-68-28-29-51-51-67-25-25-66-25-25-20-59-17-12-69-70-66-71-72-72-73-74-75-59-76-60-61-45-62
	8	564	1-3-4-5-63-11-10-64-8-48-65-66-14-25-67-14-67-21-28-68-28-21-52-29-51-51-67-25-25-20-59-17-12-69-70-66-71-72-72-73-74-75-59-76-60-61-45-62
	9	554	1-3-4-5-63-11-10-64-8-48-65-66-14-25-67-14-67-21-28-68-28-21-52-29-51-51-67-25-25-20-59-17-12-69-70-66-71-72-72-73-74-75-59-76-60-61-122
	10	628	1-3-4-5-63-11-10-10-92-11-8-9-11-48-49-32-50-29-51-28-24-32-55-32-24-32-21-48-52-29-51-51-24-32-31-15-51-25-30-55-66-14-59-24-75-59-165-60-61-45-62
	11	629	1-3-4-5-63-11-10-10-92-11-8-9-11-48-49-32-50-29-51-28-24-32-55-32-24-21-48-52-29-51-51-24-32-31-15-51-25-30-55-66-14-59-24-75-59-49-60-61-45-62
	12	717	1-3-4-5-63-11-10-10-92-11-8-9-11-48-49-32-50-29-51-28-24-32-55-32-24-21-48-52-29-51-51-24-32-31-15-51-25-30-55-66-14-59-24-75-59-49-60-61-45-98
	13	613	1-3-4-158-159-4-99-63-11-10-64-11-10-64-10-10-10-48-24-32-117-67-21-29-51-51-160-90-161-90-21-28-162-28-21-29-51-51-51-55-67-31-15-163-19-30-55-66-14-57-55-75-59-49-60-61-45-62
	47	H7920	1-3-4-99-63-11-10-64-64-10-10-10-92-10-8-9-9-11-48-49-32-29-51-54-14-32-55-32-55-180-21-52-29-51-51-14-32-31-15-25-30-32-25-58-59-67-84-20-75-30-84-85-84-86-59-76-60-87-42-43-88-45-62
	14	582	1-3-4-99-63-11-10-64-10-10-10-92-10-8-9-9-11-48-49-32-29-51-54-14-32-55-32-55-32-21-52-29-51-51-14-32-31-15-25-30-32-25-58-59-67-84-20-75-30-84-85-84-86-59-145-60-87-42-43-88-45-62
	48	H9502	1-3-4-99-63-11-10-64-10-10-10-92-10-8-9-9-11-48-49-32-29-51-54-14-32-55-32-55-32-21-52-29-51-51-24-67-31-15-25-30-32-25-58-59-67-84-20-75-30-84-85-84-86-59-76-60-87-42-43-88-45-62
	15	535-3	1-3-4-99-63-11-10-64-10-10-10-92-10-8-9-9-11-48-49-32-29-51-54-14-32-55-32-55-32-21-52-29-51-51-14-32-31-15-25-30-32-25-58-59-67-84-20-75-30-84-85-84-86-59-76-60-87-42-43-88-45-62
		618	1-3-4-99-63-11-10-64-10-10-10-92-10-8-9-9-11-48-49-32-29-51-54-14-32-55-32-55-32-21-52-29-51-51-14-32-31-15-25-30-32-25-58-59-67-84-20-75-30-84-85-84-86-59-76-60-87-42-43-88-45-62
		619	1-3-4-99-63-11-10-64-10-10-10-92-10-8-9-9-11-48-49-32-29-51-54-14-32-55-32-55-32-21-52-29-51-51-14-32-31-15-25-30-32-25-58-59-67-84-20-75-30-84-85-84-86-59-76-60-87-42-43-88-45-62
		623	1-3-4-99-63-11-10-64-10-10-10-92-10-8-9-9-11-48-49-32-29-51-54-14-32-55-32-55-

			32-21-52-29-51-51-14-32-31-15-25-30-32-25-58-59-67-84-20-75-30-84-85-84-86-59-76-60-87-42-43-88-45-62
		635	1-3-4-99-63-11-10-64-10-10-10-92-10-8-9-9-11-48-49-32-29-51-54-14-32-55-32-55-32-21-52-29-51-51-14-32-31-15-25-30-32-25-58-59-67-84-20-75-30-84-85-84-86-59-76-60-87-42-43-88-45-62
		N315	1-3-4-99-63-11-10-64-10-10-10-92-10-8-9-9-11-48-49-32-29-51-54-14-32-55-32-55-32-21-52-29-51-51-14-32-31-15-25-30-32-25-58-59-67-84-20-75-30-84-85-84-86-59-76-60-87-42-43-88-45-62
		H6556	1-3-4-99-63-11-10-64-10-10-10-92-10-8-9-9-11-48-49-32-29-51-54-14-32-55-32-55-32-21-52-29-51-51-14-32-31-15-25-30-32-25-58-59-67-84-20-75-30-84-85-84-86-59-76-60-87-42-43-88-45-62
	49	Mu50	1-3-4-99-63-11-10-64-10-10-10-92-10-8-9-9-11-48-49-32-29-51-54-14-32-14-32-31-15-25-30-32-25-58-59-67-84-20-75-30-84-85-84-86-59-76-60-87-42-43-88-45-62
		Mu3	1-3-4-99-63-11-10-64-10-10-10-92-10-8-9-9-11-48-49-32-29-51-54-14-32-14-32-31-15-25-30-32-25-58-59-67-84-20-75-30-84-85-84-86-59-76-60-87-42-43-88-45-62
	50	H13199	1-3-4-185-63-11-10-64-10-10-10-92-10-8-9-9-11-48-49-32-29-51-54-14-32-28-32-25-58-59-67-84-20-75-30-84-85-84-86-59-76-60-87-42-43-88-45-62
	16	543	1-3-4-99-63-11-10-64-10-10-10-92-10-8-9-9-11-48-49-32-29-51-54-14-32-28-32-25-58-59-67-84-20-75-30-84-85-84-86-59-76-60-87-42-43-88-45-62
		JH9	1-3-4-99-63-11-10-64-10-10-10-92-10-8-9-9-11-48-49-32-29-51-54-14-32-28-32-25-58-59-67-84-20-75-30-84-85-84-86-59-76-60-87-42-43-88-45-62
		JH1	1-3-4-99-63-11-10-64-10-10-10-92-10-8-9-9-11-48-49-32-29-51-54-14-32-28-32-25-58-59-67-84-20-75-30-84-85-84-86-59-76-60-87-42-43-88-45-62
		04-02981	1-3-4-99-63-11-10-64-10-10-10-92-10-8-9-9-11-48-49-32-29-51-54-14-32-28-32-25-58-59-67-84-20-75-30-84-85-84-86-59-76-60-87-42-43-88-45-62
		H9140	1-3-4-99-63-11-10-64-10-10-10-92-10-8-9-9-11-48-49-32-29-51-54-14-32-28-32-25-58-59-67-84-20-75-30-84-85-84-86-59-76-60-87-42-43-88-45-62
	51	H7639	1-3-4-91-63-8-10-11-11-10-92-10-92-9-11-11-51-24-58-81-58-30-7-67-93-80-67-25-94-25-20-66-24-66-55-94-25-20-66-24-66-55-66-79-24-30-32-25-95-24-66-96-97-67-25-82-84-46
	17	523-5	1-3-4-91-63-8-10-11-11-10-92-10-92-9-11-51-24-58-81-58-30-7-67-93-80-67-25-94-25-20-66-24-66-55-94-25-20-66-24-66-55-66-79-24-30-32-25-95-24-66-96-97-67-25-82-84-46
		594	1-3-4-91-63-8-10-11-11-10-92-10-92-9-11-51-24-58-81-58-30-7-67-93-80-67-25-94-25-20-66-24-66-55-94-25-20-66-24-66-55-66-79-24-30-32-25-95-24-66-96-97-67-25-82-84-46
	18	672-2	1-3-4-91-63-8-10-11-11-10-92-10-92-9-11-51-24-58-81-58-30-7-67-93-80-67-25-94-25-20-66-24-66-55-66-79-24-30-32-25-95-24-66-96-97-67-25-82-84-46
2	19	547-3	1-47-5-4-11-10-48-49-32-50-29-51-30-14-32-50-52-29-109-110-95-14-32-29-51-54-

			24-32-55-32-24-32-31-52-52-55-56-55-57-55-58-59-49-60-61-45-62
	20	521-2	89-47-5-4-11-10-48-49-32-50-29-51-30-14-32-50-52-29-53-54-14-32-29-51-54-24-32-55-32-24-32-31-52-52-55-56-55-57-55-58-59-49-60-61-90-62
	21	565	1-47-5-4-11-10-48-49-32-50-29-51-30-14-32-50-52-29-53-54-14-32-29-51-30-24-32-55-32-24-32-31-52-52-55-56-55-57-55-58-59-49-60-61-45-62
	22	535-2	1-47-5-4-11-10-48-49-32-50-29-51-30-14-32-50-52-29-53-54-14-32-29-51-54-24-32-55-32-24-32-31-52-52-55-56-55-57-55-58-59-49-60-61-45-98
	23	20-5	1-47-5-4-11-10-48-49-32-50-29-51-30-14-32-50-52-29-53-54-14-32-29-51-54-24-32-55-32-24-32-31-52-52-55-56-55-57-55-58-59-49-60-61-45-62
	24	540	100-47-5-4-11-10-10-92-9-11-101-9-9-11-101-49-32-102-51-30-24-30-25-103-25-24-80-24-32-21-55-67-21-28-104-30-84-30-11-105-7-55-106-83-7-55-24-107-108-84-61-45-62
	25	566	1-47-5-4-11-10-10-92-92-10-8-9-9-11-131-49-32-102-51-30-24-30-25-103-25-24-80-24-32-21-55-67-21-28-104-30-84-30-11-105-7-55-106-83-7-55-24-107-108-84-61-45-62
		597	1-47-5-4-11-10-10-92-92-10-8-9-9-11-131-49-32-102-51-30-24-30-25-103-25-24-80-24-32-21-55-67-21-28-104-30-84-30-11-105-7-55-106-83-7-55-24-107-108-84-61-45-62
		H13911	1-47-5-4-11-10-10-92-92-10-8-9-9-11-131-49-32-102-51-30-24-30-25-103-25-24-80-24-32-21-55-67-21-28-104-30-84-30-11-105-7-55-106-83-7-55-24-107-108-84-61-45-62
	26	627	1-47-5-4-11-10-10-92-92-10-8-9-9-11-131-49-32-102-51-30-30-25-24-80-24-32-21-55-67-21-28-104-30-84-30-11-105-7-55-106-83-7-55-24-107-164-84-61-45-62
	27	681-2	1-47-5-167-11-10-168-10-11-11-11-11-11-8-10-9-11-20-51-169-95-7-83-94-25-20-71-163-19-170-55-19-170-171-172-83-173-174-74-36-37-128-118-119-59-76-128-81-175-59-76-128-81-175-176-177-42-43-88-45-46
	28	664	1-47-5-4-11-10-8-10-48-10-8-10-48-49-32-50-29-51-30-14-32-50-52-29-53-54-14-32-29-51-54-24-32-55-32-24-32-31-52-52-24-56-55-57-55-58-59-49-60-166-45-62
3	52	MRSA252	1-2-3-4-5-63-9-10-11-10-11-10-11-40-40-146-75-30-75-15-25-66-55-80-147-51-39-73-148-83-149-19-73-149-24-19-81-67-19-15-179-15-150-84-67-29-94-31-22-66-55-151-31-22-66-152-80-30-84-67-141-75-59-153-42-154-155-156-157
	29	592	1-2-3-4-5-63-9-10-11-10-11-10-11-40-40-146-75-30-75-15-25-66-55-80-147-51-39-73-148-83-149-27-73-149-24-19-81-19-15-150-84-67-29-94-31-22-66-55-151-31-22-66-152-80-30-84-67-141-75-59-153-42-154-155-156-157
	30	20	1-2-3-4-5-6-7-8-9-10-6-10-11-8-12-13-14-15-16-12-17-18-19-20-21-22-13-14-15-16-12-17-18-19-20-21-23-24-25-26-27-15-28-29-30-29-11-31-28-32-33-34-14-35-36-37-11-38-32-39-37-40-41-42-43-44-45-46
		547-4	1-2-3-4-5-6-7-8-9-10-6-10-11-8-12-13-14-15-16-12-17-18-19-20-21-22-13-14-15-16-12-17-18-19-20-21-23-24-25-26-27-15-28-29-30-29-11-31-28-32-33-34-14-35-36-37-

			11-38-32-39-37-40-41-42-43-44-45-46
	31	579	1-2-3-4-5-6-9-9-11-40-40-40-40-11-11-40-40-135-8-75-136-95-75-136-95-67-25-66-55-67-51-137-138-139-25-75-85-140-24-15-141-15-140-20-127-30-59-38-60-141-54-59-38-60-141-119-142-143-61-45-144
4	32	584	1-2-3-4-5-6-11-40-11-8-75-123-75-15-83-15-25-66-55-67-73-24-83-67-73-24-31-19-15-130-10-81-32-28-124-11-125-86-28-126-15-19-94-82-51-127-30-59-38-60-55-64-41-42-43-128-117-27-62
		589	1-2-3-4-5-6-11-40-11-8-75-123-75-15-83-15-25-66-55-67-73-24-83-67-73-24-31-19-15-130-10-81-32-28-124-11-125-86-28-126-15-19-94-82-51-127-30-59-38-60-55-64-41-42-43-128-117-27-62
		599	1-2-3-4-5-6-11-40-11-8-75-123-75-15-83-15-25-66-55-67-73-24-83-67-73-24-31-19-15-130-10-81-32-28-124-11-125-86-28-126-15-19-94-82-51-127-30-59-38-60-55-64-41-42-43-128-117-27-62
	33	643	1-2-3-4-5-6-11-40-11-8-75-123-75-15-83-15-25-66-55-67-73-24-83-67-73-24-31-19-15-130-10-81-32-28-124-11-125-86-28-126-15-19-94-82-51-104-30-59-38-60-55-64-41-42-43-128-117-27-62
	34	558	1-2-3-4-5-6-11-40-11-8-75-123-75-15-83-15-25-66-55-67-73-24-83-67-73-24-31-19-15-9-10-81-32-28-124-11-125-86-28-126-15-19-94-82-51-127-30-59-38-60-55-64-41-42-43-128-117-27-62
	53	H6606	1-2-3-4-5-6-11-75-123-75-15-83-15-25-66-55-67-73-24-83-67-73-24-31-19-15-130-10-81-32-30-124-11-125-86-28-126-15-27-94-82-51-127-30-59-38-60-55-64-41-42-43-128-117-27-62
	35	560	1-2-3-4-129-6-11-40-11-8-83-15-25-66-55-67-73-24-83-67-73-24-31-19-15-130-10-81-32-28-124-11-125-86-28-19-94-82-51-104-30-59-38-60-55-64-41-42-43-128-117-27-62
	36	657	1-2-3-4-5-6-11-40-11-8-75-123-75-15-83-15-25-66-55-67-73-24-83-67-73-24-31-19-15-130-10-81-32-28-124-11-125-86-28-126-15-19-94-82-51-127-30-59-38-60-117-27-62
	54	H13717	1-2-3-4-5-182-32-28-124-11-125-86-29-28-126-15-19-94-82-51-127-30-59-38-60-55-64-41-42-43-128-117-183-74-184-112-27-62
5	37	507	1-77-4-5-78-8-8-8-11-11-11-67-79-17-80-73-73-51-24-15-25-24-67-24-67-15-19-80-30-81-67-24-66-24-82-83-58-11-67-84-20-75-30-84-85-84-86-59-76-60-87-42-43-88-45-62
		512	1-77-4-5-78-8-8-8-11-11-11-67-79-17-80-73-73-51-24-15-25-24-67-24-67-15-19-80-30-81-67-24-66-24-82-83-58-11-67-84-20-75-30-84-85-84-86-59-76-60-87-42-43-88-45-62
	38	577	1-77-4-5-78-8-8-8-11-11-11-67-15-17-80-73-73-51-24-15-25-24-67-24-67-15-19-80-30-81-67-24-66-24-82-83-58-11-67-84-20-75-30-84-85-84-86-59-76-60-87-42-43-88-45-62

6	39	553	100-47-5-8-111-5-10-8-112-5-8-112-5-32-55-113-114-27-7-31-28-32-52-55-19-95-115-116-21-52-10-117-66-36-37-11-118-119-59-76-10-117-56-59-41-120-121-46
