# Supplementary material for: Evolutionary Analyses of Staphylococcus aureus Identify Genetic Relationships between Nasal Carriage and Clinical Isolates
Source: PLoS One. 2011 Jan 21;6(1):e16426. doi: 10.1371/journal.pone.0016426 (PMC3025037; doi:10.1371/journal.pone.0016426)
Supplement: Table S6 — Repeat profiles for clfB. (PDF) [file pone.0016426.s008.pdf]

Table S6. Repeat profiles for *clfB*

Lineage	Haplotype	Sample	Numeric Profile
1	1	20	1-2-3-4-5-5-6-6-7-8-4-5-6-7-8-7-9-10-6-11-11-10-12-7-13-14-15-12-13-16-17-18-7-15-10-19-20-14-17
		535	1-2-3-4-5-5-6-6-7-8-4-5-6-7-8-7-9-10-6-11-11-10-12-7-13-14-15-12-13-16-17-18-7-15-10-19-20-14-17
		547-4	1-2-3-4-5-5-6-6-7-8-4-5-6-7-8-7-9-10-6-11-11-10-12-7-13-14-15-12-13-16-17-18-7-15-10-19-20-14-17
	2	565	1-2-3-4-5-5-6-6-6-7-8-4-10-12-7-13-14-15-12-13-16-17-18-7-15-10-19-20-14-17
2	3	672-2	21-2-34-35-36-11-35-37-14-14-14-36-11-37-26-27-28-25-25-29-11-11-32-23-14-23-23-32-14-23-14-38-11-39-14-36-11-40-29-32-40-41-6
	4	30	21-2-34-35-36-11-35-37-14-14-14-36-11-37-26-27-28-25-25-29-11-11-23-14-23-23-32-14-23-14-38-11-39-14-36-11-40-29-32-40-41-6
		USA300_FPR3757	21-2-34-35-36-11-35-37-14-14-14-36-11-37-26-27-28-25-25-29-11-11-23-14-23-23-32-14-23-14-38-11-39-14-36-11-40-29-32-40-41-6
		USA300_TCH1516	21-2-34-35-36-11-35-37-14-14-14-36-11-37-26-27-28-25-25-29-11-11-23-14-23-23-32-14-23-14-38-11-39-14-36-11-40-29-32-40-41-6
	5	554	21-2-34-35-36-11-35-37-14-14-14-36-11-37-26-27-28-25-25-29-11-11-32-23-35-23-23-32-14-23-14-38-11-39-14-36-11-40-29-40-41-6
	45	COL	21-2-34-35-36-11-35-37-14-14-14-36-11-37-26-27-28-25-25-11-11-32-23-14-23-23-32-14-23-14-38-11-39-14-36-11-40-29-32-40-41-6
		Newman	21-2-34-35-36-11-35-37-14-14-14-36-11-37-26-27-28-25-25-11-11-32-23-14-23-23-32-14-23-14-38-11-39-14-36-11-40-29-32-40-41-6
	6	637	21-2-34-35-36-11-35-37-14-14-14-36-11-37-26-27-28-25-29-11-11-23-14-23-23-32-14-23-14-38-11-39-14-36-11-40-29-32-40-41-6
	7	517	21-2-34-35-36-11-35-37-25-14-14-36-11-37-26-27-28-25-25-29-11-11-23-23-32-14-23-14-38-11-39-14-36-11-40-29-32-40-41-6
		521-3	21-2-34-35-36-11-35-37-25-14-14-36-11-37-26-27-28-25-25-29-11-11-23-23-32-14-23-14-38-11-39-14-36-11-40-29-32-40-41-6
	8	523-5	21-2-34-37-14-14-14-36-11-37-26-27-28-25-25-29-11-11-32-23-14-23-32-14-23-14-38-11-39-14-36-11-40-29-32-40-41-6
		594	21-2-34-37-14-14-14-36-11-37-26-27-28-25-25-29-11-11-32-23-14-23-32-14-23-14-38-11-39-14-36-11-40-29-32-40-41-6
	46	H6556	21-2-34-35-36-11-35-37-14-14-35-36-11-37-26-27-28-25-25-29-7-11-31-32-23-14-23-31-25-23-14-38-11-39-11-40-45
	9	605	21-2-35-36-11-35-37-14-14-14-36-11-37-26-27-28-25-25-23-14-23-23-32-14-23-14-38-11-39-14-36-11-40-29-40-41-6
	10	535-3	21-2-34-35-36-11-35-37-14-14-35-36-11-37-26-27-28-25-25-29-11-23-32-23-14-23-23-25-23-14-38-11-39-11-40-45

		618	21-2-34-35-36-11-35-37-14-14-35-36-11-37-26-27-28-25-25-29-11-23-32-23-14-23-23-25-23-14-38-11-39-11-40-45
		619	21-2-34-35-36-11-35-37-14-14-35-36-11-37-26-27-28-25-25-29-11-23-32-23-14-23-23-25-23-14-38-11-39-11-40-45
		635	21-2-34-35-36-11-35-37-14-14-35-36-11-37-26-27-28-25-25-29-11-23-32-23-14-23-23-25-23-14-38-11-39-11-40-45
		H7920	21-2-34-35-36-11-35-37-14-14-35-36-11-37-26-27-28-25-25-29-11-23-32-23-14-23-23-25-23-14-38-11-39-11-40-45
		H9502	21-2-34-35-36-11-35-37-14-14-35-36-11-37-26-27-28-25-25-29-11-23-32-23-14-23-23-25-23-14-38-11-39-11-40-45
	11	543	21-2-34-35-36-11-35-37-14-14-35-36-11-37-26-27-28-25-25-29-11-31-32-23-14-23-23-25-23-14-38-11-39-11-40-45
		N315	21-2-34-35-36-11-35-37-14-14-35-36-11-37-26-27-28-25-25-29-11-31-32-23-14-23-23-25-23-14-38-11-39-11-40-45
		Mu50	21-2-34-35-36-11-35-37-14-14-35-36-11-37-26-27-28-25-25-29-11-31-32-23-14-23-23-25-23-14-38-11-39-11-40-45
		Mu3	21-2-34-35-36-11-35-37-14-14-35-36-11-37-26-27-28-25-25-29-11-31-32-23-14-23-23-25-23-14-38-11-39-11-40-45
		04-02981	21-2-34-35-36-11-35-37-14-14-35-36-11-37-26-27-28-25-25-29-11-31-32-23-14-23-23-25-23-14-38-11-39-11-40-45
		H9140	21-2-34-35-36-11-35-37-14-14-35-36-11-37-26-27-28-25-25-29-11-31-32-23-14-23-23-25-23-14-38-11-39-11-40-45
		H13199	21-2-34-35-36-11-35-37-14-14-35-36-11-37-26-27-28-25-25-29-11-31-32-23-14-23-23-25-23-14-38-11-39-11-40-45
	47	JH9	21-2-34-35-36-11-37-37-14-14-35-36-11-37-26-27-28-25-25-29-11-31-32-23-14-23-23-25-23-14-38-11-39-11-40-45
		JH1	21-2-34-35-36-11-37-37-14-14-35-36-11-37-26-27-28-25-25-29-11-31-32-23-14-23-23-25-23-14-38-11-39-11-40-45
	12	547-3	21-2-34-35-36-11-35-37-14-14-35-36-11-37-26-27-28-25-25-29-11-31-37-23-14-23-23-25-23-14-38-11-39-11-40-45
		582	21-2-34-35-36-11-35-37-14-14-35-36-11-37-26-27-28-25-25-29-11-31-37-23-14-23-23-25-23-14-38-11-39-11-40-45
	48	NCTC8325	21-2-34-35-36-11-37-26-27-28-25-25-29-11-11-32-23-14-23-23-32-14-23-14-38-11-39-14-36-11-40-29-32-40-41-6
	49	H7639	21-2-34-35-36-11-35-21-14-37-43-27-44-106-23-30-31-32-23-14-29-29-29-32-31-32-14-29-23-25-19-7-33-14-17
	13	623	21-2-34-35-36-11-35-37-14-14-35-36-11-37-26-27-28-25-25-29-11-31-32-23-14-23-23-25-23-14-38-11-40-45
3	50	TW20	45-2-24-45-6-46-27-47-14-7-48-30-49-50-47-47-46-27-17-44-21-56-7-45-45-47-7-52-

			7-53-21-41-20-50-16-42-14-33-33-7-29-13-55-7-17
	14	535-2	45-2-24-45-6-46-27-47-14-7-48-30-49-50-47-47-46-27-17-44-21-56-7-41-45-47-7-52-7-53-21-41-20-50-16-42-14-33-13-7-29-13-55-7-17
		547	45-2-24-45-6-46-27-47-14-7-48-30-49-50-47-47-46-27-17-44-21-56-7-41-45-47-7-52-7-53-21-41-20-50-16-42-14-33-13-7-29-13-55-7-17
	51	H7681	45-2-24-45-6-46-27-47-14-7-48-30-49-50-47-47-46-27-17-44-21-56-7-45-45-47-7-52-7-53-21-41-20-50-16-42-14-33-53-29-13-55-7-17
	15	720	45-24-45-6-46-27-47-14-7-48-30-49-50-46-27-17-44-21-56-7-41-45-47-7-52-7-53-21-41-20-50-16-42-14-33-15-7-29-13-55-7-17
	52	H7051	45-2-24-45-6-46-27-47-47-47-46-27-17-44-21-56-7-45-45-47-7-52-7-53-21-41-20-50-16-42-14-33-33-7-29-13-55-7-17
		H7951	45-2-24-45-6-46-27-47-47-47-46-27-17-44-21-56-7-45-45-47-7-52-7-53-21-41-20-50-16-42-14-33-33-7-29-13-55-7-17
	16	524	45-2-24-45-6-46-27-47-14-7-48-30-49-50-47-47-51-7-52-7-53-21-41-20-50-16-42-14-42-14-33-54-7-29-13-55-7-17
	17	592	45-2-24-45-6-46-27-47-5-14-7-48-30-49-50-47-47-51-7-52-7-53-21-41-20-50-16-42-14-33-54-7-29-13-55-7-17
	18	512	45-2-24-45-6-6-46-27-47-14-7-48-30-49-50-47-47-51-7-52-53-21-41-20-50-16-42-14-33-54-7-29-13-55-7-17
	19	521	45-2-24-45-6-46-27-47-14-7-48-30-49-50-47-47-51-7-52-7-53-21-41-20-50-16-42-14-33-54-7-29-13-55-7-17
		563	45-2-24-45-6-46-27-47-14-7-48-30-49-50-47-47-51-7-52-7-53-21-41-20-50-16-42-14-33-54-7-29-13-55-7-17
		636	45-2-24-45-6-46-27-47-14-7-48-30-49-50-47-47-51-7-52-7-53-21-41-20-50-16-42-14-33-54-7-29-13-55-7-17
		662	45-2-24-45-6-46-27-47-14-7-48-30-49-50-47-47-51-7-52-7-53-21-41-20-50-16-42-14-33-54-7-29-13-55-7-17
	20	599	45-2-24-45-46-27-47-14-7-48-30-49-50-47-47-51-7-52-7-53-21-41-20-50-16-42-14-33-54-7-29-13-55-7-17
	21	651	45-2-24-45-6-46-27-47-14-53-48-30-49-50-47-47-51-7-52-7-53-21-41-20-50-16-42-14-33-54-7-29-42-7-17
	53	MRSA252	45-2-24-45-6-46-27-47-14-7-48-14-50-47-47-51-7-52-7-53-21-41-20-50-16-42-14-33-54-7-29-13-55-7-17
	22	531	45-2-24-45-6-46-27-47-14-7-48-30-49-50-47-47-51-7-52-7-53-21-41-20-50-16-42-14-33-54-7-29-42-7-17
		607	45-2-24-45-6-46-27-47-14-7-48-30-49-50-47-47-51-7-52-7-53-21-41-20-50-16-42-14-33-54-7-29-42-7-17
		608	45-2-24-45-6-46-27-47-14-7-48-30-49-50-47-47-51-7-52-7-53-21-41-20-50-16-42-14-33-54-7-29-42-7-17

	23	710	45-2-24-45-6-46-27-47-14-7-48-30-49-50-47-47-51-7-53-21-41-20-50-16-42-14-33-54-7-29-42-7-17
4	24	540	9-24-20-23-24-9-42-14-37-43-27-44-29-23-29-37-43-5-44-29-23-32-43-27-32-14-29-7-23-23-35-29-30-29-30-23-35-29-11-40-45
	25	627	9-24-20-23-24-9-42-14-37-43-27-44-29-23-30-29-37-43-5-44-29-23-32-43-27-32-14-29-7-23-23-35-29-30-29-30-23-11-40-45
	26	507	9-24-20-23-24-9-42-14-37-43-27-44-29-23-30-29-37-43-5-44-29-23-32-43-27-32-14-29-7-29-30-29-30-23-35-29-11-40-45
	27	597	9-24-20-23-24-9-42-14-37-43-27-44-29-23-30-29-37-43-5-44-29-23-32-43-27-32-14-29-7-23-23-35-29-11-40-45
	28	628	9-24-20-23-24-9-42-14-29-23-32-43-27-32-14-29-7-23-23-35-29-11-40-45
		629	9-24-20-23-24-9-42-14-29-23-32-43-27-32-14-29-7-23-23-35-29-11-40-45
		717	9-24-20-23-24-9-42-14-29-23-32-43-27-32-14-29-7-23-23-35-29-11-40-45
	29	566	9-24-20-23-24-9-42-14-29-7-11-35-29-30-29-30-23-25-29-11-40-45
	54	H13911	9-24-20-23-24-9-42-14-37-43-27-44-29-23-30-29-37-43-5-44-29-23-32-43-27-32-14-29-7-23-23-35-29-30-29-30-23-35-29-11-40-45
	30	714	9-24-20-23-24-9-42-14-37-43-27-44-29-23-30-29-37-43-5-44-29-23-32-43-27-29-29-7-23-23-35-29-30-29-30-23-35-29-11-40-107
	55	MSSA476	9-24-20-23-24-9-42-14-37-43-27-44-29-23-30-29-37-43-5-44-29-23-32-43-27-25-29-7-23-23-35-29-30-29-108-23-35-29-11-40-45
	56	MW2	9-24-20-23-24-9-42-14-37-43-27-44-29-23-30-29-37-43-5-44-29-23-32-43-27-25-29-7-23-23-35-29-30-29-30-23-35-29-11-40-45
	57	H9779	9-24-9-42-14-37-43-27-44-29-23-30-29-37-43-5-44-29-23-32-43-27-25-29-7-23-23-35-29-30-29-30-23-35-29-11-40-45
5	31	558	7-63-64-7-63-64-7-21-16-17-65-66-67-14-20-20-15-14-48-7-9-68-13-14-48-7-7-20-15-16-20-15-14-9-68-13-14-48-7-20-15-14-52-48-7-20-13-14-41-15-38-17
	32	560	7-63-64-7-63-64-7-21-16-17-65-69-14-20-20-15-14-48-7-9-68-13-14-48-70-7-20-15-14-9-68-17
	33	657	7-63-64-7-63-64-7-21-16-17-65-69-14-20-20-15-14-48-7-9-68-13-14-48-70-7-20-15-16-20-15-14-9-68-13-105-48-7-20-15-14-52-48-7-20-13-14-41-15-38-17
	58	H6606	7-63-64-7-63-64-7-21-16-17-65-69-14-20-20-15-14-48-7-9-68-13-14-48-7-7-20-15-16-20-15-14-9-68-13-14-48-7-20-15-14-52-48-7-20-13-14-41-15-38-17
	34	589	7-63-64-7-63-64-7-21-68-15-14-48-7-9-68-13-14-48-70-7-20-15-16-20-15-16-20-15-14-9-68-13-14-48-7-20-15-14-52-48-7-20-13-14-41-15-38-17
	59	H13717	7-63-64-7-21-14-44-20-20-15-14-48-7-9-68-13-14-48-70-7-20-15-16-52-48-7-20-15-16-41-13-7-20-15-16-52-48-7-20-15-16-41-109-7-32
	35	643	7-63-64-7-63-64-7-21-16-17-65-69-14-20-15-14-48-7-9-68-13-14-48-70-7-20-15-14-20-15-14-9-68-17
6	36	664	21-22-20-23-24-9-25-26-27-28-25-25-29-11-11-32-23-14-23-30-31-32-23-14-29-29-

			32-23-32-23-25-23-14-29-23-25-19-7-33-14-17
	37	20-5	21-22-20-23-24-9-25-26-27-28-25-25-29-11-30-14-23-30-31-32-23-7-29-29-29-29-32-23-32-23-25-23-14-29-23-25-19-7-33-14-17
		547-2	21-22-20-23-24-9-25-26-27-28-25-25-29-11-30-14-23-30-31-32-23-7-29-29-29-29-32-23-32-23-25-23-14-29-23-25-19-7-33-14-17
7	38	577	21-2-34-35-36-11-35-37-14-14-14-36-14-37-43-11-24-20-21-92-21-14-37-43-27-44-93-23-30-31-32-23-14-29-29-29-94-95-29-32-31-32-23-25-23-14-29-23-25-19-7-33-14-17
	39	681-2	43-11-24-20-21-92-36-33-14-37-43-27-44-106-23-31-25-23-14-29-32-29-29-32-23-32-23-32-23-25-23-14-29-23-25-19-7-33-14-17
8	40	613	27-2-24-52-103-32-104-21-22-53-15-14-37-43-23-41-35-36-37-13-14-37-43-14-29-7-29-23-35-35-11-13-14-37-43-27-17-23-25-31-14-29-25-19-31-25-30-52-47-32-23-25-17
9	41	574	1-2-24-41-52-9-88-31-52-21-14-42-11-14-17-88-31-52-17-88-31-52-21-45-46-27-14-42-14-33-13-7-41-13-7-41-16-89-29-17-90-91-30
10	42	564	71-72-24-41-68-44-47-36-73-17-74-75-76-77-17-52-75-14-78-30-52-75-79-25-80-75-81-82-83-32-81-84-27-32-85-11-86-11-87
11	43	579	1-96-20-50-52-33-97-33-47-5-6-46-98-14-99-29-17-15-3-21-6-100-101-20-52-33-102-9-6-14-20-33-6-7-22-14-9-38-17
12	44	553	1-2-3-57-4-7-58-30-59-30-60-11-13-61-44-41-62-12-7-13-14-13-11-15-13-16-17-18-7-13-62-19-20-14-17
