# Supplementary material for: Evolutionary Analyses of Staphylococcus aureus Identify Genetic Relationships between Nasal Carriage and Clinical Isolates
Source: PLoS One. 2011 Jan 21;6(1):e16426. doi: 10.1371/journal.pone.0016426 (PMC3025037; doi:10.1371/journal.pone.0016426)
Supplement: Text S1 — Repeat profiling program for clfA. (PDF) [file pone.0016426.s009.pdf]

The clf repeat profiling program, gensh, was written using the C++ programming language and designed to convert DNA sequences in the serine-aspartic acid repeat domain (R domain) into numeric profiles. When a series of DNA sequences are entered into the program, the R domain start site is identified and used as the beginning of the first repeat unit. Beginning with the first repeat, consecutive 18 bp segments of DNA are then analyzed. All uniqe repeat units are given a different number in the sequence in which they are encountered (all identical repeats are given the same number). When identifying repeat units, 18 bp segments are analyzed; however, 12 bp repeats are identified by the presence of "TCN" at bases 13-15. When "TCN" is present at bases 13-15, the program treats this a the start of a new repeat unit and identifies the previous repeat as only 12 bp. This process continues until the stop sequence, which identifies the end of the R domain, is encountered. Note that because the program arbitrarily numbers repeat units in the order in which they are encountered, no inference can be made as to the degree of nucleotide similarity between different numbers (e.g. repeat number 1 is not necessarily more similar to repeat number 2 than any other repeat number). After the program is complete, two files will be generated. One contains a log of the numbered repeat sequences that were identified throughout the complete dataset while the other is a file containing the sample names and their numeric repeat profiles. Note that the source code below is only for clfA DNA sequences. DNA sequences from clfA and clfB cannot be combined and analyzed in the same data set. For DNA sequences of clfB R domains, refer to Supplementary file 2.

To use the program, gensh:

compile using g++
example: g++ gensh.cpp -o gensh

This creates a gensh file that can be run by:
./gensh INFILE OUTFILE1 OUTFILE2

where the INFILE is a fasta formatted DNA file containing clf R domain DNA sequences and OUTFILEs are the files that the program produces. OUTFILE1 is a tab deliminated file containing all repeat units identified in the dataset along with their repeat numbers. OUTFILE2 is also a tab deliminated file and contains the sample names and their numeric repeat profiles.

```
#include <iostream>
#include <fstream>
#include <list>
#include <vector>

using namespace std;

//Global Variables
fstream INFILE;
fstream OUTFILE1;
fstream OUTFILE2;

typedef struct{
  string Segment;
  int Number;
}TChunk;

vector<string> SeqName;

//Function Declarations
void ReadInput(list<string> &SeqList);
void CreateOutput(list<string> SeqList);
int FindChunk(vector<TChunk> list, string chunk);

int main(int argc, char* argv[]) {

   /*cout << "argc = " << argc << endl;
   for(int i = 0; i < argc; i++)
      cout << "argv[" << i << "] = " << argv[i] << endl;*/

   if(argc < 4 || argc > 4){
      cout << "Program input as follows:" << endl;
      cout << "\t./gensh INFILE OUTFILE1 OUTFILE2\n" << endl;
      return 0;
   }


   INFILE.open(argv[1], ios::in);
```

```
   OUTFILE1.open(argv[2], ios::out);
   OUTFILE2.open(argv[3], ios::out);

   string FirstSeq;
   list<string> SeqList;

   ReadInput(SeqList);
   CreateOutput(SeqList);

   return 0;
}

void ReadInput(list<string> &SeqList){
   //Hold the sequence name just in case we need it later...
   string Line;
   string CompleteSeq;
   bool SequenceStored = false;

   if (INFILE.is_open()) {
      /* ok, proceed with output */
      cout << "WOHO, lets collect some strings!" << endl;

      while( !INFILE.eof() ){

         //Read in the Sequence Name
         getline(INFILE, Line);

         switch (Line[0]){
            case '>':
               //Handle the Line as a name
               SeqName.push_back(Line.substr(1, Line.size()-1));
               if(SequenceStored){
                  //cout << "SEQ = " << CompleteSeq << endl;
                  SeqList.push_back(CompleteSeq);
                  SequenceStored = false;
               }
               CompleteSeq.clear();
               break;
            default:
               //Keep adding up the String...
               SequenceStored = true;
               if(Line[Line.size()-1] == '\n')
                  CompleteSeq += Line.substr(0, Line.size()-1);
               else
                  CompleteSeq += Line.substr(0, Line.size());
               break;
         }
      }
      SeqList.push_back(CompleteSeq);
      INFILE.close();
   }
}

void CreateOutput(list<string> SeqList)
{
   bool terminated = false;
   vector<TChunk> UniqueSeq;
   vector<TChunk> Outfile;
   //Temporary Chunk that will be pushed onto the list it is not a duplicate
   TChunk tmp;
   string chunk;
   int count = 0;
   for (list<string>::iterator it = SeqList.begin(); it != SeqList.end(); it++)
   {
      string seq = *it;
      //cout << seq << endl;

      for(int i=0; i<seq.size()-2; i++)
      {
         if(seq.substr(i,3) == "GAT")
```

```
    {
       if(seq.substr(i+3, 2) == "TC" && (seq.substr(i+6, 3) == "GAC"
          || seq.substr(i+6, 3) == "GAT"))
       {
          cout << "Found a MATCH!" << endl;

          //From the newly found chunk to the end of the complete seq.
          seq = seq.substr(i+3, seq.size());

          //Find the terminating seq, if not found skip out of everything.
          terminated = false;
          for(int j=0; j<seq.size(); j++)
          {
             if(seq.substr(j,2) == "TC" && seq.substr(j+3, 9) == "AACAATAAT")
             {
                terminated = true;
                seq = seq.substr(0,j);
             }
          }
          if(terminated)
          {
   OUTFILE2 << SeqName.at(count) << '\t';
             //cout << "TERMINATED" << endl;
             //cout << seq << endl;
             while(seq.size() >= 12)
             {
                //location of the chunk, -1 if not found in the list.
                int chunk_location;

                //Grab the 18 character chunk and check to see if it matches specs
                if(seq.size() < 18)
                {
                   chunk = seq;
                   seq.clear();
                }
                else
                {
                   chunk = seq.substr(0, 18);
                //cout << "CHUNK = " << chunk << endl;
                //If the chunk has "TC" in pos 13-14, copy only the first 12 and
                //restart chunks from TC...
                if(chunk.substr(12, 2) == "TC")
                {
                   //cout << "TCN found :" << endl;
                   chunk = chunk.substr(0,12);
                   seq = seq.substr(12, seq.size());
                }
                else
                {
                   //cout << "TCN Not found : " << endl;
                   seq = seq.substr(18, seq.size());
                }
                }
                chunk_location = FindChunk(UniqueSeq, chunk);

                if(chunk_location >= 0)
                {
                   OUTFILE2 << (chunk_location+1);
                   //UniqueSeq.at(chunk_location).Number += 1;
                }
                else
                {
                   TChunk tmp = {chunk, 1};
                   UniqueSeq.push_back(tmp);
                   OUTFILE2 << UniqueSeq.size();
                }
                if(seq.size() >= 12)
                   OUTFILE2 << '-';
             }
          }
```

```
                else
                {
                   OUTFILE2 << SeqName.at(count) << '\t' << "ERROR" << '\n';
                }
                break;
             }
          }
       }
       if(terminated)
          OUTFILE2 << '\n';
    /*   //Output everything to a file yo...
       OUTFILE1 << SeqName.at(count) << '\n';
       OUTFILE2 << SeqName.at(count) << '\t';
       for(int i=0; i<UniqueSeq.size(); i++)
       {
          OUTFILE1 << (i+1) << ' ' << UniqueSeq.at(i).Segment << '\n';
          for(int j=0; j<UniqueSeq.at(i).Number; j++)
             OUTFILE2 << (i+1) << '-';
       }
       OUTFILE1 << '\n';
       OUTFILE2 << '\n';*/
       count++;
    }

    for(int i=0; i<UniqueSeq.size(); i++)
    {
       OUTFILE1 << (i+1) << ' ' << UniqueSeq.at(i).Segment << '\n';
    }
}

int FindChunk(vector<TChunk> list, string chunk)
{
    for(int i=0; i<list.size(); i++)
    {
       string tmp = list.at(i).Segment;
       if(tmp == chunk)
          return i;
    }
    return -1;
}
```
