# Supplementary material for: Evolutionary Analyses of Staphylococcus aureus Identify Genetic Relationships between Nasal Carriage and Clinical Isolates
Source: PLoS One. 2011 Jan 21;6(1):e16426. doi: 10.1371/journal.pone.0016426 (PMC3025037; doi:10.1371/journal.pone.0016426)
Supplement: Text S2 — Repeat profiling program for clfB. (PDF) [file pone.0016426.s010.pdf]

This clf repeat profiling program is intended to be used with clfB R domain DNA sequences only.  It is similar to that for clfA repeat profiling; however, the stop site has been changed for use with locus clfB.  Refer to Supplementary file 1 for a detailed description of what the program does and how it works.

To use the program, gensh:

compile using g++
example:  g++ gensh.cpp -o gensh

This creates a gensh file that can be run by:
./gensh INFILE OUTFILE1 OUTFILE2

where the INFILE is a fasta formatted DNA file containing clf R domain DNA sequences and OUTFILEs are the files that the program produces.  OUTFILE1 is a tab deliminated file containing all repeat units identified in the dataset along with their repeat numbers.  OUTFILE2 is also a tab deliminated file and contains the sample names and their numeric repeat profiles.

```
#include <iostream>
#include <fstream>
#include <list>
#include <vector>

using namespace std;

//Global Variables
fstream INFILE;
fstream OUTFILE1;
fstream OUTFILE2;

typedef struct{
  string Segment;
  int Number;
}TChunk;

vector<string> SeqName;

//Function Declarations
void ReadInput(list<string> &SeqList);
void CreateOutput(list<string> SeqList);
int FindChunk(vector<TChunk> list, string chunk);

int main(int argc, char* argv[]) {

   /*cout << "argc = " << argc << endl;
   for(int i = 0; i < argc; i++)
      cout << "argv[" << i << "] = " << argv[i] << endl;*/

   if(argc < 4 || argc > 4){
      cout << "Program input as follows:" << endl;
      cout << "\t./gensh INFILE OUTFILE1 OUTFILE2\n" << endl;
      return 0;
   }


   INFILE.open(argv[1], ios::in);
   OUTFILE1.open(argv[2], ios::out);
   OUTFILE2.open(argv[3], ios::out);

   string FirstSeq;
   list<string> SeqList;

   ReadInput(SeqList);
   CreateOutput(SeqList);

   return 0;
}

void ReadInput(list<string> &SeqList){
   //Hold the sequence name just in case we need it later...
```

```
   string Line;
   string CompleteSeq;
   bool SequenceStored = false;

   if (INFILE.is_open()) {
      /* ok, proceed with output */
      cout << "WOHO, lets collect some strings!" << endl;

      while( !INFILE.eof() ){

         //Read in the Sequence Name
         getline(INFILE, Line);

         switch (Line[0]){
            case '>':
               //Handle the Line as a name
               SeqName.push_back(Line.substr(1, Line.size()-1));
               if(SequenceStored){
                  //cout << "SEQ = " << CompleteSeq << endl;
                  SeqList.push_back(CompleteSeq);
                  SequenceStored = false;
               }
               CompleteSeq.clear();
               break;
            default:
               //Keep adding up the String...
               SequenceStored = true;
               if(Line[Line.size()-1] == '\n')
                  CompleteSeq += Line.substr(0, Line.size()-1);
               else
                  CompleteSeq += Line.substr(0, Line.size());
               break;
         }
      }
      SeqList.push_back(CompleteSeq);
      INFILE.close();
   }
}

void CreateOutput(list<string> SeqList)
{
   bool terminated = false;
   vector<TChunk> UniqueSeq;
   vector<TChunk> Outfile;
   //Temporary Chunk that will be pushed onto the list it is not a duplicate
   TChunk tmp;
   string chunk;
   int count = 0;
   for (list<string>::iterator it = SeqList.begin(); it != SeqList.end(); it++)
   {
      string seq = *it;
      //cout << seq << endl;

      for(int i=0; i<seq.size()-2; i++)
      {
         if(seq.substr(i,3) == "GAT")
         {
            if(seq.substr(i+3, 2) == "TC" && (seq.substr(i+6, 3) == "GAC"
               || seq.substr(i+6, 3) == "GAT"))
            {
               cout << "Found a MATCH!" << endl;

               //From the newly found chunk to the end of the complete seq.
               seq = seq.substr(i+3, seq.size());

               //Find the terminating seq, if not found skip out of everything.
               terminated = false;
               for(int j=0; j<seq.size(); j++)
               {
                  if(seq.substr(j,2) == "TC" && seq.substr(j+3, 9) == "GATTCAAGA")
```

```
            {
               terminated = true;
               seq = seq.substr(0,j);
            }
         }
         if(terminated)
         {
   OUTFILE2 << SeqName.at(count) << '\t';
            //cout << "TERMINATED" << endl;
            //cout << seq << endl;
            while(seq.size() >= 12)
            {
               //location of the chunk, -1 if not found in the list.
               int chunk_location;

               //Grab the 18 character chunk and check to see if it matches specs
               if(seq.size() < 18)
               {
                  chunk = seq;
                  seq.clear();
               }
               else
               {
                  chunk = seq.substr(0, 18);
               //cout << "CHUNK = " << chunk << endl;
               //If the chunk has "TC" in pos 13-14, copy only the first 12 and
               //restart chunks from TC...
               if(chunk.substr(12, 2) == "TC")
               {
                  //cout << "TCN found :" << endl;
                  chunk = chunk.substr(0,12);
                  seq = seq.substr(12, seq.size());
               }
               else
               {
                  //cout << "TCN Not found : " << endl;
                  seq = seq.substr(18, seq.size());
               }
               }
               chunk_location = FindChunk(UniqueSeq, chunk);

               if(chunk_location >= 0)
               {
                  OUTFILE2 << (chunk_location+1);
                  //UniqueSeq.at(chunk_location).Number += 1;
               }
               else
               {
                  TChunk tmp = {chunk, 1};
                  UniqueSeq.push_back(tmp);
                  OUTFILE2 << UniqueSeq.size();
               }
               if(seq.size() >= 12)
                  OUTFILE2 << '-';
            }
         }
         else
         {
            OUTFILE2 << SeqName.at(count) << '\t' << "ERROR" << '\n';
         }
         break;
      }
   }
}
if(terminated)
   OUTFILE2 << '\n';
/*   //Output everything to a file yo...
   OUTFILE1 << SeqName.at(count) << '\n';
   OUTFILE2 << SeqName.at(count) << '\t';
   for(int i=0; i<UniqueSeq.size(); i++)
```

```
      {
         OUTFILE1 << (i+1) << ' ' << UniqueSeq.at(i).Segment << '\n';
         for(int j=0; j<UniqueSeq.at(i).Number; j++)
            OUTFILE2 << (i+1) << '-';
      }
      OUTFILE1 << '\n';
      OUTFILE2 << '\n';*/
      count++;
   }

   for(int i=0; i<UniqueSeq.size(); i++)
   {
      OUTFILE1 << (i+1) << ' ' << UniqueSeq.at(i).Segment << '\n';
   }
}

int FindChunk(vector<TChunk> list, string chunk)
{
   for(int i=0; i<list.size(); i++)
   {
      string tmp = list.at(i).Segment;
      if(tmp == chunk)
         return i;
   }
   return -1;
}
```
