# Supplementary material for: Evolutionary Analyses of Staphylococcus aureus Identify Genetic Relationships between Nasal Carriage and Clinical Isolates
Source: PLoS One. 2011 Jan 21;6(1):e16426. doi: 10.1371/journal.pone.0016426 (PMC3025037; doi:10.1371/journal.pone.0016426)
Supplement: Text S3 — clf color-coded repeat generator. (PDF) [file pone.0016426.s011.pdf]

The program, wgraph, was written using the C++ programming language and designed to convert numeric clf R domain profiles (such as those created by the accompanying program, gensh) into color-coded representations. When a file containing numeric repeat profiles is entered into the program, a color coded visual representation is generated. Identical numbers are colored the same throughout the data set so repeats containing identical DNA sequence are colored the same. When initiated, the program automatically reads an input file containing hexadecimal colors that it then uses to generate the output. The output can be saved by using the print screen command. The size of the individual boxes on the graph can be adjusted as per the user's preference by changing the S_WIDTH and S_HEIGHT parameters.

To use the program, wgraph:

compile using g++
example: g++ wgraph.cpp -o wgraph

This creates a wgraph file that can be run by:
./wgraph INFILE.dat

where the INFILE is a .dat file containing numeric profiles for clf R domain DNA sequences.

```
#include <GL/glut.h>
#include <iostream>
#include <fstream>
#include <string>
#include <stdlib.h>
#include <string.h>
#include <stdio.h>
#include <vector>

using namespace std;

#define WINDOW_X 1000
#define WINDOW_Y 1000
#define S_WIDTH 12
#define S_HEIGHT 44

void displayCB(void);
void SetPenColorHex(unsigned long color);
void DrawFillBox(double x0, double y0, double x1, double y1);
int xtoi(const char* xs, unsigned long* result);

int ypos = WINDOW_Y - 10;
int xpos = 10;

void displayCB(void)            /* function called whenever redisplay needed */
{
   glClear(GL_COLOR_BUFFER_BIT);                /* clear the display */


   //Open and read the hex datafile...
   //unsigned long hex;
   string line;
   vector<string> HexList;
   ifstream myfile ("hex.dat");
   if (myfile.is_open())
   {
      while (! myfile.eof() )
      {
         getline (myfile,line);
         HexList.push_back(line);
         /*xtoi(line.c_str(), &hex);
         //cout << hex << endl;
         SetPenColorHex(hex);
         //cout << line << endl;
         DrawFillBox(xpos, ypos-S_HEIGHT, xpos+S_WIDTH, ypos);
         xpos += (S_WIDTH+1);
         if(xpos > (WINDOW_X - 20))
         {
            xpos = 10;
            cout << "BEFORE: " << S_HEIGHT << endl;
            ypos = ypos - S_HEIGHT - 10;
```

```
            cout << "Y POS: " << ypos << endl;
         }*/

      }
      myfile.close();
   }
   else cout << "Unable to open  hex file" << endl;
   //Read the Datfile and print this...
   myfile.open("infile.dat");
   if (myfile.is_open())
   {
      int pos;
      string Name;
      while (! myfile.eof() )
      {
         getline(myfile,line);
         if(line.length() > 0)
         {
            pos = line.find("\t");
            Name = line.substr(0,pos);
            cout << "Name: " << Name << " Size=" << line.length() <<  endl;
            line = line.substr(pos+1, line.length());
            cout << "Line: " << line << endl;

            int found=line.find_first_of("-");
            unsigned long hex;
            while (found!=string::npos)
            {
               int val = atoi(line.substr(0,found).c_str());
               xtoi(HexList.at(val).c_str(), &hex);
               SetPenColorHex(hex);
               DrawFillBox(xpos, ypos-S_HEIGHT, xpos+S_WIDTH, ypos);
               xpos += (S_WIDTH+1);
               line = line.substr(found+1, line.length());
               found=line.find_first_of("-");
            }
            int val = atoi(line.c_str());
            xtoi(HexList.at(val).c_str(), &hex);
            SetPenColorHex(hex);
            DrawFillBox(xpos, ypos-S_HEIGHT, xpos+S_WIDTH, ypos);

            xpos = 10;
            ypos = ypos - S_HEIGHT - 10;
         }
      }
      myfile.close();
   }


   //DrawFillBox(20, 20, 40, 70);
   glFlush();                           /* Complete any pending operations */
}

void keyCB(unsigned char key, int x, int y)     /* called on key press */
{
  if( key == 'q' ) exit(0);
}


int main(int argc, char *argv[])
{
  int win;

  glutInit(&argc, argv);                /* initialize GLUT system */

  glutInitDisplayMode(GLUT_RGB);
  glutInitWindowSize(WINDOW_X,WINDOW_Y);        /* width=400pixels height=500pixels */
  win = glutCreateWindow("Triangle");   /* create window */

  /* from this point on the current window is win */
```

```
  glClearColor(1.0,1.0,1.0,1.0);        /* set background to black */
  gluOrtho2D(0,WINDOW_X,0,WINDOW_Y);            /* how object is mapped to window */
  glutDisplayFunc(displayCB);           /* set window's display callback */
  glutKeyboardFunc(keyCB);              /* set window's key callback */

  glutMainLoop();                       /* start processing events... */

  /* execution never reaches this point */

  return 0;
}

void SetPenColor(double red, double green, double blue)
{
        glColor3d(red,green,blue);
}

void SetPenColorHex(unsigned long color)
{
   SetPenColor((color >> 16) / 256.0,
              (color >> 8 & 0xFF) / 256.0,
              (color & 0xFF) / 256.0);
}

void DrawFillBox(double x0, double y0, double x1, double y1)
{
   glBegin(GL_POLYGON);                 // Draw a connected line from
      glVertex2d(x0,y0);                                // corner to
      glVertex2d(x1,y0);                                // lcorner to
      glVertex2d(x1,y1);                                // corner to
      glVertex2d(x0,y1);                                // corner to
      glVertex2d(x0,y0);                                // corner,
   glEnd();                                             // then stop--we're finished.
}

// Converts a hexadecimal string to integer
int xtoi(const char* xs, unsigned long* result)
{
   size_t szlen = strlen(xs);
   int i, xv, fact;

   if (szlen > 0)
   {
      // Converting more than 32bit hexadecimal value?
      if (szlen>8) return 2; // exit

      // Begin conversion here
      *result = 0;
      fact = 1;

      // Run until no more character to convert
      for(i=szlen-1; i>=0 ;i--)
      {
         if (isxdigit(*(xs+i)))
         {
            if (*(xs+i)>=97)
            {
               xv = ( *(xs+i) - 97) + 10;
            }
            else if ( *(xs+i) >= 65)
            {
               xv = (*(xs+i) - 65) + 10;
            }
            else
            {
               xv = *(xs+i) - 48;
            }
            *result += (xv * fact);
            fact *= 16;
```

```
        }
        else
        {
        // Conversion was abnormally terminated
        // by non hexadecimal digit, hence
        // returning only the converted with
        // an error value 4 (illegal hex character)
           return 4;
        }
     }
  }

  // Nothing to convert
  return 1;
}
```
